# Scoping review of precision child and youth mental health research: dwelling in possibility

**DOI:** 10.3389/fpsyt.2025.1691548

**Published:** 2026-02-09

**Authors:** Joonsoo Sean Lyeo, Angelica Blais, Paula Cloutier, Addo Boafo, Aroldo Dargél, Amanda Helleman, Tanya Tanya, Esperance Kashala-Abotnes, Christina Honeywell, Kathleen Pajer

**Affiliations:** 1 Children's Hospital of Eastern Ontario (CHEO) Research Institute, Ottawa, ON, Canada; 2 Department of Psychiatry, University of Ottawa Medical School, Ottawa, ON, Canada; 3 The Ottawa Hospital, Ottawa, ON, Canada

**Keywords:** precision mental health, precision behavioural health, precision psychiatry, children, adolescents, youth

## Abstract

**Introduction:**

Precision child and youth mental health (PCYMH) offers a promising array of tools and methodologies to address the intensifying burden of mental health challenges in child and youth populations. However, the current state of PCYMH research requires better characterization. To this end, we conducted a scoping review aiming to provide a ‘lay of the literature’ for this emerging field.

**Methods:**

Following the Joanna Briggs Institute methodology for scoping reviews, we searched PubMed and Embase for PCYMH studies from January 1, 1980 to November 30, 2023, updating the search on November 1, 2024. The final dataset comprised 124 publications, summarized with descriptive quantitative analysis and qualitative content analysis.

**Results:**

Quantitative analyses revealed that 48% (60/124) of studies had been published between 2020 and 2024, with the majority (51% (63/124)) studying populations in the U.S. Most studies were observational in design. Content analysis revealed four categories of PCYMH research focus: (1) Biomarkers (68% (84/124)); (2) Non-Biological Markers (17% (22/124)); (3) Implementation of PCYMH Interventions (14% (17/124)); and (4) Predictive Algorithms (5% (6/124)). PCYMH tools were underutilized and infrequently combined. Studies producing multimodal profiles of participants, e.g., using neuroimaging, genetics, digital health data, and lifestyle data were scarce. No study used reporting guidelines.

**Discussion:**

Our findings indicate that this body of research is still in its infancy. We highlight opportunities to advance the study of PCYMH and provide recommendations to support the maturation of this new field.

## Introduction

Child and youth mental health (CYMH) problems have intensified and are now considered a public health crisis (1). Nearly 15% of youth ages 10–19 years have experienced a mental illness, accounting for 13% of the global burden of disease within this population (1). As many as 1 in 5 people are diagnosed with a mental illness before the age of 25, with 70% experiencing their first symptoms before the age of 18 (2). Emerging evidence demonstrates a rising prevalence and severity of mental illness, particularly anxiety and mood disorders, as reflected in increasing rates of mental health service utilization and psychotropic medication use (3). The burden of CYMH problems is projected to intensify in the coming years, driven by the rise of social media consumption, enduring consequences of the COVID-19 pandemic, heightened experiences of loneliness and social isolation, and mounting concerns about a future shaped by global instability and climate change (4, 5).

In recent years, a growing proportion of CYMH cases have been identified as having ‘complex mental health needs’, requiring higher intensity services and more frequent care with sustained involvement from CYMH agencies (6). Emergency departments (EDs) are becoming the default sites for CYMH care (7), with ED visits surpassing increases in CYMH outpatient visits (8). This is problematic as EDs have limited capacity to provide such care (8, 9). In addition, nearly a quarter of CYMH patients re-visit EDs for more mental healthcare within six months (9, 10). This suggests that many CYMH patients cannot get their needs met, highlighting the inability of current healthcare systems to provide treatment in an effective or timely manner (11).

The limitations of current CYMH care have received increased scrutiny (12). For instance, Bickman et al. (13) called into question the utility, reliability, and validity of psychiatric diagnoses in CYMH care, determining in a three-part study of affective and behavior disorders of children and youth that: few CYMH outcomes were diagnosed more consistently than a random selection of symptoms; there was low diagnostic inter-rater agreement between parents, youth, and clinicians; and that comorbidities posed a significant barrier to clinician-based diagnoses (13). Insel further questioned the *status quo* of CYMH care, highlighting the need to pivot away from reliance on behavioral symptoms, the predominant method for diagnosis of CYMH disorders, to instead create a neurodevelopmental framework (14).

It is clear that transformation of CYMH care and the research that drives it is necessary. The Precision Child and Youth Mental Health (PCYMH) paradigm has the potential to accomplish this (15, 16). Such a transformation will not be easy and must not discard the advances made in evidence-based care (17, 18), but the standard ‘one size fits all’ approach, doesn't work for all (18).

Furthermore, the growing popularity of the PCYMH paradigm is, in part, a response to the problems stemming for questionable validity of CYMH diagnoses, concerns about underlying heterogeneity in patients labeled with the current nomenclature, and the difficulty of applying statistical mean-based results from randomized controlled trials (RCTs) to the individual needs of children and youth (15, 19).

Advances in PCYMH have already yielded several promising avenues for tailoring mental health services (15). For instance, at the Children’s Hospital of Eastern Ontario (CHEO), the creation of a participatory logic model demonstrated how the synthesis of implementation science and artificial intelligence (AI) data science can be used to plan a PCYMH research and clinical care transformation program (20). Another example is the development of a clinical decision support system by a Norwegian research team, that has shown how big data analytics, and the electronic health record (EHR) can be used to create an Individualized Digital Decision Assist System (IDDEAS) to enhance precision and timeliness of medical decisions via clinician decision-making support based on targeted clinical knowledge and patient health information (21, 22).

However, many developments in the growing field of PCYMH research remain fragmented and siloed. For PCYMH methods to be smoothly integrated into the wider corpus of CYMH care and research, it is essential to catalogue the work done to date. Such efforts are the first step towards establishing consistent definitions and terminology within the field and may help guide funders on strategic allocation of their resources.

To this end, we conducted a scoping review aiming to provide a ‘lay of the literature’ review of the emerging field of PCYMH. Our goal was to provide a snapshot of current scientific work published under the rubric of PCYMH or its synonyms and make recommendations for future research.

## Methods

We used the most recent version of the Joanna Briggs Institute (JBI) methodology to conduct scoping reviews (23). The review was organized into 5 stages: (1) specification of a research question; (2) systematic retrieval of studies from the scientific literature; (3) screening them for relevance to the research question; (4) extracting information about the studies; (5) conducting descriptive quantitative and qualitative content analysis of retained studies; and (6) synthesizing these data into a final summary.

### Research question and search strategy

We formulated the research question using the Population, Concept, Context (PCC) framework (23): What are the characteristics or features of all published research studies involving precision child and youth mental health? This exercise facilitated development of the search strategy, guided creation of inclusion and exclusion criteria, and structured the data collection.

The review took a broad view, rather than focusing on a specific disorder or method. Our study was structured according to the Preferred Reporting Items for Systematic Reviews and Meta-analysis (PRISMA-ScR) (see **Supplementary Table 1** for framework components' locations in the paper) (23). We searched PubMed and Embase, in consultation with an in-house librarian. Together, these two databases provide extensive coverage of medical and biomedical academic literature from the past eight decades, with each database comprising over 25 million records. Because the concept of precision medicine had its roots earlier than 1999 (24), the usual landmark date cited, we conducted our search from January 1, 1980 to November 30, 2023, updating it on November 1, 2024.

The search strategy was mapped onto the following three domains with synonyms: (1) precision health (e.g., ‘precision medicine’, ‘precision health’, ‘personalized medicine’); (2) child and youth (e.g., ‘child’, ‘adolescent’, ‘youth’); and (3) mental health (e.g., ‘mental health’, ‘behavioral health’, ‘child psychiatry’). Because child and youth mental health research often takes place in pediatric settings, we set the age range at 0 to <18 years. Relevant keywords and synonyms for each conceptual construct were joined using ‘OR’, with ‘AND’ being used to join the three constructs into a single search strategy. The search strategy and results for PubMed and Embase are presented in **Supplementary Tables 2**, **3** respectively.

### Inclusion and exclusion criteria

PCYMH research was defined as studies with aims or goals that addressed precision diagnosis, treatment, prognosis, or prevention of a mental health, psychiatric, or behavioral health condition based on differences in individuals’ biological characteristics, lifestyle, and environment, in addition to symptoms. Studies were included if they: (1) investigated the topic of precision mental health; (2) had a primary study population between the ages of 0 and up to 18 years old or, if in a study of adults and children/youth, separate findings on the 0-18-year-olds were provided; (3) were published in English; and 4) had “precision” or one of its synonyms in the title or abstract. Documents and publications not presenting published original research were excluded, e.g., conference abstracts, commentaries, literature reviews, meta-analyses, book chapters, and reports.

Prior to starting the screening process, inclusion and exclusion criteria were iteratively refined by JSL and KP through a series of consultations with other members of the research team. The research team pointed out that our initial inclusion criteria of “0–18 years of age” could be interpreted as those “up to” or “including the 18^th^ year of life (which would make them 19)”. They gave examples from other studies showing that many papers with this age range often were included in studies of adults, without any separate results for the 18- year-olds. Therefore, this inclusion criterion was adjusted to specify “up to, but not including, the 18^th^ year of life”.

### Screening and data extraction

Covidence (25) was used to process papers retrieved from the search. Screening comprised two steps, both of which were carried out following the eligibility criteria by pairs of ten raters. Ratings were conducted blind to ratings by others. The first step was title and abstract screening. Articles remaining eligible after this step were next subjected to full-text review, again conducted by pairs of raters blind to each other’s work. For both steps, reviewer discrepancies were resolved through deliberation between KP and JSL.

All papers still meeting criteria after full-text review underwent final data extraction by two blinded raters, using the rubric in **Table 1**. In addition to data more commonly collected on study characteristics, we also collected information on “type of PCYMH study” (defined by the aim(s) or objective(s)), each study’s use of PCYMH tools, e.g., use of big data, AI, omics (19, 26),and whether or not a reporting guideline was used to structure the paper.

**Table 1:** Data extraction rubric.

Characteristic	Definition and values
Title	
First author	
Year of publication	
Study goals/aim(s)	Summary as pertains to PCYMH
Type of PCYMH study	Biomarker Non-Biological Marker PCYMH Implementation Intervention AI Predictive Algorithm
Primary clinical focus	Aggression Anxiety Attention deficit hyperactivity disorder Autism spectrum disorder Bipolar disorder Conduct disorder Major depressive disorder Obsessive–compulsive disorder Oppositional defiant disorder Post-traumatic stress disorder Psychosis Self-harm Suicidality
Geographic setting	Country; if multi-national study, country of main site
Sample size	
Power analysis	Yes No
Sample age range	
Sample gender composition	Male (n) Female (n) Other (n)
Data sources (all that apply)	Administrative data Clinical assessments Interviews Lab results Surveys or questionnaires
Use of a reporting guideline	Yes No
Study Design	Case-control Case series Cohort Cross-sectional Non-randomized control trial Randomized control trial Qualitative N-of -1
Primary statistical analyses	Brief summary
PCYMH tools (all that apply)	Use of Big data, including EHRs Digital health data AI and Machine learning AI and Multivariable profiling Omics of any type Pharmacogenetics Virtual data sample No PCYMH tools used
Key study findings	Brief summary
Conclusion	Brief summary

### Data analysis

Quantitative data were summarized with descriptive statistics. Qualitative analysis, particularly looking for patterns in clinical disorders or problems, type of PCYMH study, and use of PCYMH tools was based on the approach to latent pattern content analysis outlined by Potter & Levine-Donnerstein (27). As per this approach, analysis comprised the following four stages: (1) decontextualization – identification of recurrent units of meaning; (2) recontextualization – organization of the data into codes; (3) categorization – grouping of codes into categories and sub-categories of shared meaning; and (4) compilation – final refinement of categories and sub-categories. This process was iterative and recursive, with the findings generated during each stage of the analysis informing the coder’s approach to subsequent stages. All steps of the qualitative analysis were independently conducted by the first and senior authors.

## Results

### Article screening

**Figure 1:**
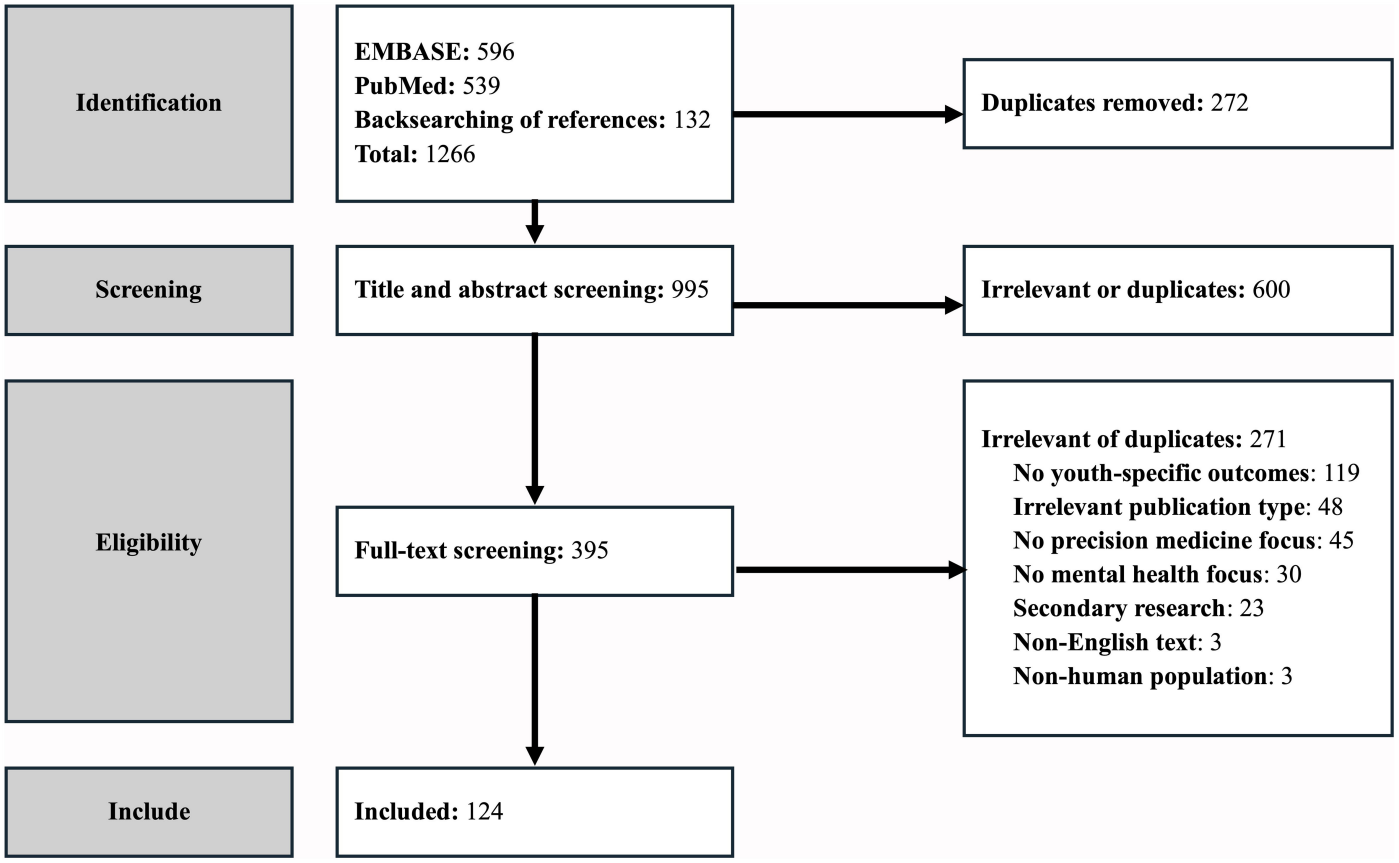
PRISMA Diagram.

The search yielded 1,266 studies (see **Figure 1** for the PRISMA diagram). Of these studies, 272 were flagged by Covidence as duplicates and removed. All automatic duplications were manually verified by the first author. The remaining 995 studies underwent title and abstract screening, during which 600 studies were dropped as not meeting criteria. Full text screening identified 271 more that did not meet criteria. The remaining 124 (28–151) articles comprised the final set for review.

### Characteristics of articles

**Figure 2:**
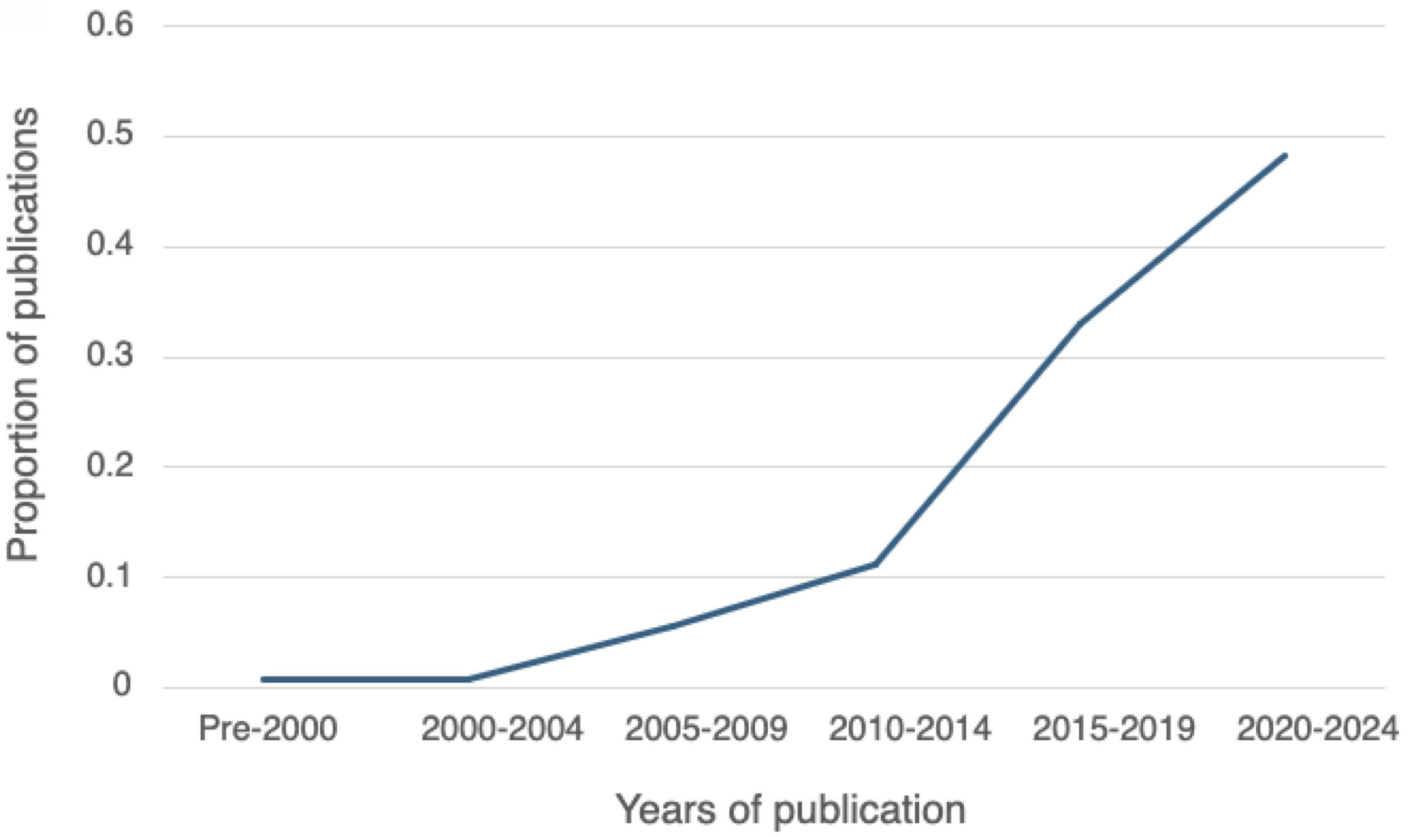
Proportion of studies by publication dates (N=124).

Dates of publication for the articles are displayed in **Figure 2** by four-year blocks of time from pre-2000 to 2024. Only one paper meeting our criteria was published before 2000. This was followed by a slow but steady rise in publication rate until an inflection point occurred between 2010-2014, signaling a sharp uptick in the publication rates every four years over the subsequent decade. The highest rate to date was between 2020-2024, during which 48% (60/124) (29–31, 35, 39, 43, 44, 48, 50, 51, 54, 59, 61–63, 66–69, 74, 76–78, 80–83, 89, 90, 93–96, 98, 99, 105, 108–113, 115, 117–119, 121, 123, 124, 128, 131–133, 137, 139, 146–150) of the entire set was published.

**Figure 3:**
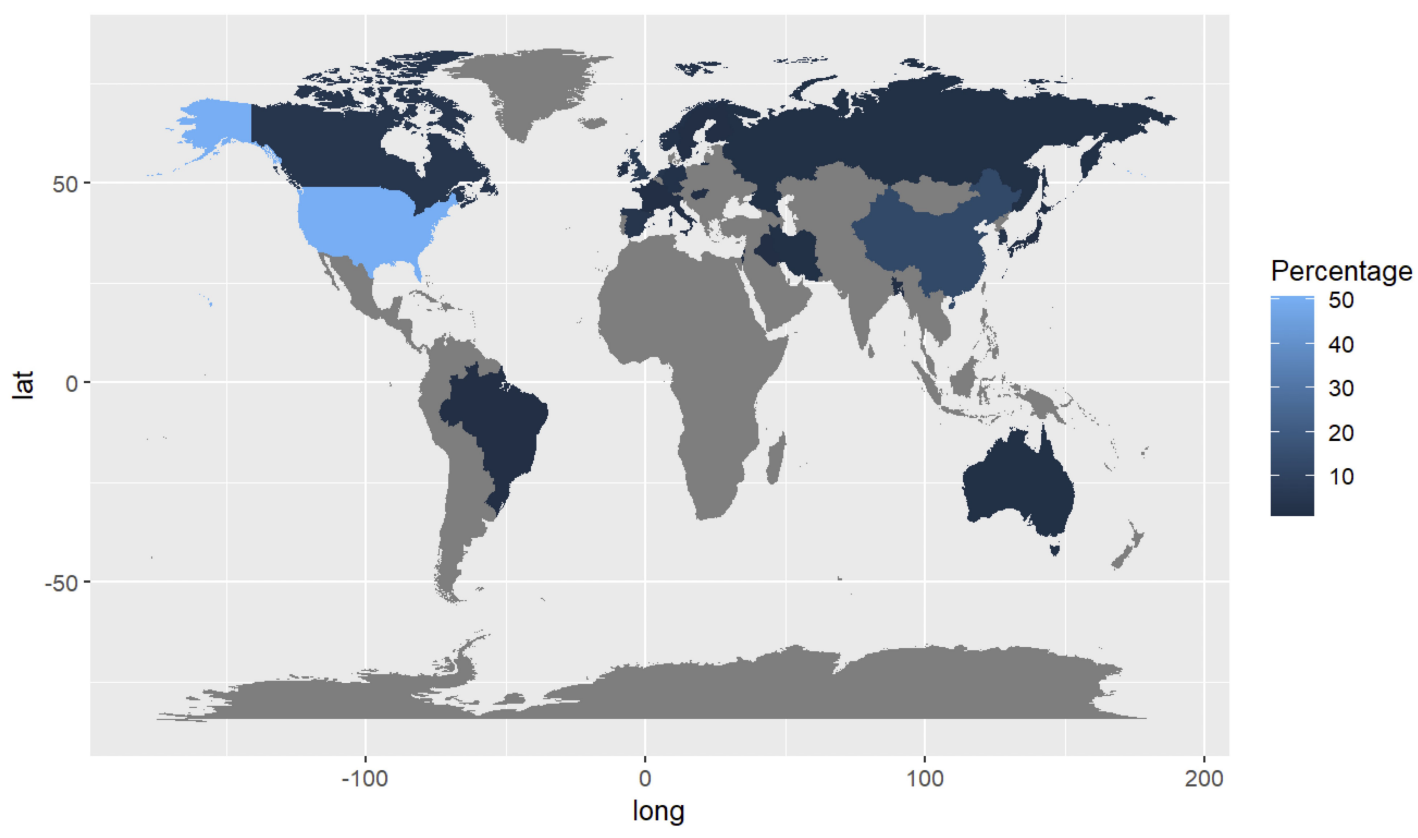
Proportion of studies by country of origin (N=124).

Articles were from 26 countries, with multi-site study papers coded according to the primary site, determined through descriptions of the study setting, the senior author’s address, and locations of funding sources. As can be seen on the map in **Figure 3**, 51% (63/124) (28, 31, 32, 35, 37, 41, 43, 45–48, 50, 52–56, 58, 59, 64, 66, 69, 70, 74–76, 80, 82–84, 88–92, 96, 97, 99, 103, 106–109, 113–117, 120, 123, 126, 132–136, 139–142, 144, 146, 150) of the papers were from the United States, with China and the UK representing 8% (10/124) (68, 87, 95, 111, 125, 137, 143, 147–149) and 6% (7/124) (86, 93, 94, 104, 122, 128, 145), respectively. The remaining countries each contributed 1–6 papers.

### Study features

None of the included studies used a reporting guideline. **Table 2** provides an overview of the studies included, organized by first author’s last name and displaying CYMH focus, type of PCYMH study as defined by aim, PCYMH tool(s), and key findings. **Supplementary Tables 4**-**7** present more details on each study.

**Table 2:** High-level summary of included studies by MH focus, type of PCYMH study, PCYMH tool(s), and key findings (N = 124) by first author’s last name.

First author (year)	MH focus	Type of PCYMH study	PCYMH tool(s)	Key findings
Biomarker studies				
Adams (2011) (28)	ASD	Identify gut flora and GI^1^ biomarkers a/w^2^ ASD^3^.	None	GI symptoms were strongly correlated with the severity of ASD.
Aggensteiner (2024) (29)	CD/ODD	Design SCL^4^ arousal-bio-feedback training to reduce aggression in CD/ODD^5^.	None	The SCL biofeedback treatment was neither superior nor inferior to the active TAU^6^.
Al-Ali (2022) (30)	ASD	Assess the clinical utility of blood OXT^7^ serum levels and receptor genotype as biomarkers of ASD.	Omics	Peripheral OXT levels and OXT receptor genetic alterations were identified as potential biomarkers of social functioning in the ASD patient setting.
Arnett (2022) (32)	ADHD	Measure the association between positive response to MPH^8^ treatment and abnormal frontal-striatal neural network excitation.	None	MPH responders demonstrated attenuated P3 amplitude relative to controls, while responders did not differ on this measure.
Arns (2008) (33)	ADHD	Investigate the predictive value of EEG^9^ phenotypes for stimulant medication effectiveness in ADHD^10^.	None	The Frontal Slow and Slowed APF and the Low Voltage EEG phenotype discriminated ADHD subjects best from controls.
Arns (2018) (34)	ADHD	Replicate and extend EEG biomarkers used to predict non-response to ADHD medication.	None	For treatment prediction, clear gender and age-group differences were found, where a low APF^11^ in male adolescents with ADHD was a/w a smaller likelihood of responding to MPH.
Baker (2021) (35)	ASD	Measure association between social motivation and neural changes from pre- to post-intervention in ASD.	None	Parent-reported social responsiveness and social skills improved in adolescents with ASD after participation in a precision medicine intervention.
Bazanova (2018) (36)	ADHD	Determine EEG and EMG^12^ biomarkers most related to the main ADHD characteristics.	None	ADHD children were characterized with decreased individual APF, alpha bandwidth and alpha amplitude suppression magnitude, as well as with increased alpha1/alpha2 ratio and scalp muscle tension.
Bernas (2018) (38)	ASD	Propose a novel MRI^13^-based ASD biomarker by analyzing temporal brain dynamics in resting-state fMRI^14^.	None	Study shows change in the coherence of temporal neurodynamics is a biomarker of ASD, and wavelet coherence-based classifiers lead to robust and replicable results which could be used as an objective ASD diagnostic tool.
Brown (2016) (41)	ADHD	Characterize the effect of CYP2D6^15^ genotype on the dose-exposure relationship for atomoxetine.	Omics	Dose-corrected ATX^16^ systemic exposure varied 29.6-fold across the study cohort. Simulated steady state profiles at the maximum US Food and Drug Administration-recommended dose suggest that most patients are unlikely to attain adequate ATX exposures.
Bruxel (2013) (42)	ADHD	Evaluate the association between a 75 T4G polymorphism and appetite reduction as a side effect of MPH in children with ADHD.	None	The G allele presented a trend for association with appetite reduction scores; however, the G allele carriers presented a higher risk for appetite reduction worsening when compared with T allele homozygotes.
Cardinale (2023) (43)	Anxiety	Disentangle cognitive control correlates of anxiety versus irritability.	None	Results of whole-brain multivariate linear models revealed that anxiety at age 15 was uniquely a/w decreased neural response to conflict across multiple regions implicated in attentional control and conflict adaptation.
Carpentieri (2023) (44)	ADHD	Search for clinical biomarkers of ADHD in CpG^17^ methylation patterns.	None	For “improving” ADHD children, CpGs 3 and 5 were methylated with CpG 2 and CpG 6; however, for “severe” ADHD children, CpGs 2 and 5 accompanied a methylated CpG 1.
Connolly (2017) (45)	MDD	Examine whether amygdala RSFC^18^ is a/w changes in MDD^19^ severity in adolescents.	None	Compared to healthy controls, depressed adolescents showed reduced amygdala-based RSFC with the dorsolateral PFC^20^ and the ventromedial PFC.
Doruk Camsari (2019) (47)	MDD	Examine baseline measures of cortical inhibition and excitability in depressed patients and characterize their longitudinal posttreatment changes.	None	Baseline, short-interval intracortical inhibition-2 was significantly reduced in depressed participants, suggesting impaired cortical inhibition compared with healthy controls.
Edmunds (2022) (48)	ADHD	Examine if comorbid ADHD and anxiety features or EEG measures of engagement moderated the extent to which children benefited from the EF^21^ training.	None	EF training improved behavioral inhibition only for children with clinically significant co-occurring ADHD features; meanwhile anxiety features, while prevalent, did not moderate EF training efficacy.
Efstathopoulos (2018) (49)	Internalizing symptoms	Examine the association between NR3C1^22^ methylation and the emergence of internalizing symptoms in childhood and adolescence.	None	NR3C1 hypermethylation was cross-sectionally a/w high score for internalizing symptoms in the whole group as well as among the female participants.
Faedda (2016) (52)	BD	Test the hypothesis that objective measures of activity, sleep, and circadian rhythms would help differentiate pediatric subjects with BD^23^ from ADHD and TD^24^ controls.	ML^25^; Digital Health Data	There were prominent group differences in several activity measures, notably mean 5 lowest hours of activity, skewness of diurnal activity, relative circadian amplitude, and vulnerability index.
Forbes (2010) (53)	MDD	Provide preliminary evidence that pretreatment reward-related brain function in the striatum and medial PFC could have relevance for predicting both final level and rate of change of clinical characteristics in adolescents with MDD.	None	Final levels of severity and anxiety symptoms were a/w pretreatment striatal reactivity, and rate of anxiety symptom reduction was a/w greater striatal reactivity and lower medial PFC reactivity.
Ford (2023) (54)	Anhedonia; MDD	Investigate symptom network patterns in adolescents from a GBA^26^ biopsychosocial perspective. Test the GBA Pathways Systems Theory relationship and investigate symptom networks for their overall associations with anhedonia and depressed mood.	Multimodal profile	The GBA perspective revealed several symptom neighbors that could expand clinical assessment, diagnosing criteria, education, and interventions for adolescents at risk for, or with, anhedonia or depressed mood: weight loss, self-worth tied to weight, difficulty sustaining attention, poor eye contact, etc.
Frazier (2016) (55)	ASD	Create an objective, eye tracking-based ASD risk index.	Multimodal profile	In both samples, the ASD risk index had high diagnostic accuracy, was strongly a/w Autism Diagnostic Observation Schedule–Second Edition severity scores, and not significantly correlated with language ability.
Frazier (2018) (56)	ASD	Develop and validate eye tracking-based measures for estimating ASD risk and quantifying ASD symptom levels.	Multimodal profile	Eye tracking measures appear to be useful quantitative, objective measures of ASD risk and ASD symptom levels.
Gassó (2014) (57)	Various mental health disorders	Evaluate the influence of CYP2D6, CYP2C9^27^ and ABCB1^28^ genotypes on the steady-state plasma concentrations of fluoxetine and its active metabolite (S)-norfluoxetine, and on the clinical improvement in patients receiving fluoxetine treatment.	Omics	Results confirm the influence of CYP2D6 genetic variants in fluoxetine pharmacokinetics and provide evidence for the potential effect of the ABCB1 genotype on the clinical improvement in children and adolescent patients treated with fluoxetine.
Ging-Jehil (2023) (59)	ADHD	Explore whether baseline cognitive processing moderated the effect of NF^29^ on improvement in the composite parent- and teacher-rated inattention score from baseline to end-treatment.	ML; Multimodal profile	Pre-treatment cognitive testing with computational modeling identified children who benefitted more from NF than control treatment for ADHD.
Griffiths (2019) (60)	ADHD	Explore differences in ERPs^30^ that are a/w noradrenergic activity—N2 and P3—in atomoxetine responders versus non-responders.	None	Responders were distinguished by significantly lower auditory oddball N2 amplitudes than both non-responders and TD controls, particularly in the right frontocentral region.
Gutiérrez-Casares (2021) (61)	ADHD	Model the efficacies of the two drugs, lisdexamfetamine and MPH, and compare them in a virtual head-to-head setting. Describe an approach to measure and compare the output results in terms of efficacy of the two medications, the molecular mechanisms triggered, and the response to ADHD management in a diverse population of virtual patients, including patients with the most common psychiatric comorbidities.	Big data; ML; Virtual population	Provided adult and pediatric-adolescent virtual populations and generated quantitative systems pharmacology models to infer the mechanism of action of lisdexamfetamine and MPH.
Hagenbeek (2020) (62)	Aggression	Examine the association of urinary metabolites and neurotransmitter ratios involved in key metabolic and neurotransmitter pathways in a cohort of twins and clinic-referred children, using biomarker panels to identify correlates of aggression.	Omics	6 biomarkers were significantly a/w childhood aggression, of which the association of O-phosphoserine, and gamma-L-glutamyl-L-alanine remained significant after multiple testing.
Hegarty (2019) (64)	ASD	Examine the relationships between structural measures of language regions in the brain and changes in language abilities following PRT^31^ in young children with ASD.	None	Level of improvement on the SLO^32^ was correlated with baseline asymmetry of the inferior frontal gyri, while the size of the left superior temporal gyri at baseline was correlated with the level of improvement on standardized parental questionnaires.
Hong (2012) (65)	ADHD	Investigate the interactions between dopamine transporter gene, dopamine D4 receptor gene, alpha-2A adrenergic receptor gene, and norepinephrine transporter gene in ADHD treatment response to MPH.	Omics	Genetic determinants of MPH response consist of both dopaminergic and noradrenergic gene polymorphisms.
Ivashchenko (2020) (67)	Psychosis	Analyze possible associations of gene polymorphisms with the effectiveness and safety of antipsychotics in adolescents with an acute psychotic episode for 14 days of treatment.	None	Established that CYP2D6 intermediate metabolizer phenotype and polymorphisms ABCB1 2677G>T/A and 3435C>T were significantly a/w a higher frequency of several ADEs^33^.
Jiang (2023) (68)	ASD	Identify and model brain-wide differences in structural connectivity using diffusion tensor imaging in young ASD and TD children.	Big data, ML	Revealed the presence of a small number of inter-regional structural connections within the brains of young children with ASD which exhibit increased FA^34^ compared to TD and negatively a/w symptom severity.
Karcher (2023) (69)	Psychosis	Examine the degree to which persistent and distressing PLEs^35^ exhibit neural metrics that show similarity to adults with chronic psychiatric and neurologic conditions.	Big data	Findings suggest that especially the persistent distressing PLEs in children were a/w neural metrics resembling those observed in adults with severe psychiatric and neurologic conditions.
Kelly (2019) (70)	ASD	Investigate blood and stool metabolomic profiles a/w the ASQ^36^ derived communication score, as a proxy for ASD risk, in children from the Vitamin D Antenatal Asthma Reduction Trial, a clinical trial of prenatal vitamin D supplementation and outcomes in pregnant women and their offspring.	Big data; Omics	Identified a number of metabolomic pathways and metabolites with biologically plausible relationships with impaired development of communication skills and with ASD risk.
Kim (2011) (71)	MDD; Anxiety	Evaluate the effects of val66met on hippocampal volume and on encoding-related hippocampal activity while considering the potential influence of childhood abuse and diagnostic status.	Omics	Val66met was found to have a significant impact on hippocampal volume independently of childhood abuse and psychiatric status.
Kim (2015) (72)	ADHD	Examine whether applying ML to pretreatment demographic, clinical, environmental, neuropsychological, neuroimaging, and genetic information can predict ADHD therapeutic response following MPH administration.	Omics;ML; Big data; Multimodal profile	Findings support an association between homozygosity for the Val allele and better response to MPH in Korean ADHD children as assessed by four different response criteria.
Kirley (2003) (73)	ADHD	Examine a dopamine transporter gene as conferring susceptibility to ADHD.	None	There is an association between the 10-repeat VNTR^37^ DAT1^38^ polymorphism and retrospectively rated MPH response.
Klimes-Dougan (2022) (74)	MDD	Evaluate if baseline structure and function of the amygdala and ACC^39^ predict response to Interpersonal Psychotherapy for Depressed Adolescents.	None	The following were a/w greater improvement to MDD symptoms: greater ACC activation during an emotion-matching task and greater amygdala-ACC resting-state functional connectivity. There was minimal evidence that brain structure predicted changes in depressive symptoms.
Klimes-Dougan (2018) (75)	Aggression	Examine the stress activation and response system to: differentiate high versus low-risk children, and to explore indicators a/w favorable intervention response.	None	Findings provide preliminary evidence that hypothalamic pituitary adrenal axis biological variables may be helpful tools for identifying children who would benefit from intervention and personalizing interventions.
Kurkinen (2023) (77)	MDD	Discover metabolic alterations in sexually or physically abused depressed adolescent psychiatric outpatients.	Big data; Omics	Revealed alterations in metabolites related to one-carbon metabolism, mitochondrial dysfunction, oxidative stress, and inflammation in depressed patients with a history of sexual or physical abuse.
Kyeong (2017) (79)	ADHD	Determine whether new ADHD clinical phenotypes can be identified based on symptom severity and IQ^40^ measurements. A second aim was to investigate whether neuroimaging findings validate identified phenotypes.	ML; Multimodal profile	Demonstrated that the use of common clinical phenotypes and an innovative unsupervised data-driven ML algorithm is an informative approach for understanding the heterogeneity of ADHD.
Latrèche (2021) (81)	ASD	Examine the relationship between social orienting and baseline clinical characteristics in young children with ASD, as well as explored the role of social orienting as a predictor of developmental change and treatment outcome.	None	Attention to face is robustly correlated with ASD symptoms, developmental skills, developmental change, and verbal gains in particular. Social orienting predicted a better treatment outcome in the context of an early and intensive intervention, paving the way toward.
Lee (2022) (83)	ADHD	Use the ABCD^41^ dataset to examine shared and non-shared neural correlates of response inhibition and error processing across distinct phenotypes of ADHD, irritability, and their co-occurrence using data-driven, latent variable modeling techniques.	None	Latent class analysis revealed four phenotypic groups based on severity of ADHD and co-occurring irritability. Group differences emerged in the neural coactivation network a/w response inhibition but not error processing.
Lewis (2016) (84)	MDD	Examine the relationship between MDD severity and TMS^42^ measures of cortical inhibition and excitability in children and adolescents	None	Preliminary results provide evidence for a relationship between MDD severity and dysfunction in GABA^43^ergic and glutamatergic cortical processes.
Lim (2013) (86)	ADHD	Apply Gaussian process classification to grey matter volumetric data to assess whether ADHD adolescents can be accurately differentiated from healthy controls based on objective, brain structure measures.	None	Discriminating grey matter patterns showed higher association between ADHD and earlier developing ventrolateral/premotor fronto-temporo-limbic.
Loo (2016) (88)	ADHD	Compare effects of MPH, guanfacine, and combination of the two on resting state EEG and determine if these a/w improvements in behavioral and cognitive functioning.	None	Revealed distinct underlying medication-related effects on neural mechanisms. The combination condition uniquely exhibited an EEG profile that was a/w improved behavioral and cognitive functioning.
Loo (2021) (89)	ADHD	Test cognitive and EEG predictors of treatment response with ADHD.	None	Those with more severe executive dysfunction are more likely to be TNS^44^ responders, show modulation of right frontal brain activity, improved/normalized EFs, and ADHD symptom reduction.
Mahjani (2021) (90)	ASD	Evaluate the frequency of pdSNVs^45^ and their impact on medical and psychiatric phenotypes relative to pdCNVs^46^.	Omics	Rare pdSNVs were more common than pdCNVs, with the combined yield of potentially damaging variation was substantial at 27%. The results provide compelling rationale for the use of high-throughput sequencing as part of routine clinical workup for ASD.
McGinnis (2019) (91)	General mental illness	Use ML to identify children with internalizing disorders using an instrumented 90-second fear induction task.	Digital health data; ML	When paired with ML, the data collected from 20 seconds of wearable sensor use during a fear induction task can be used to identify young children with internalizing disorders with a high level of accuracy, sensitivity, and specificity.
McGough (2006) (92)	ADHD	Explore genetic moderators of symptom reduction and side effects in MPH-treated children with ADHD.	None	Emerging evidence suggests the potential for optimizing ADHD medications on the basis of individual genetics.
Meng (2021) (95)	ASD	Identify differential proteins in the urinary proteome between ASD and non-ASD children aged 3–7 years.	Omics	118 differential proteins were identified in the urine between ASD and non-ASD children, with cadherin-related family member 5 and vacuolar protein sorting-associated protein 4B showing the best discriminative ability.
Michelini (2023) (96)	ADHD	Investigate pretreatment clinical and EEG profiles as predictors of treatment outcome in children randomized to MPH and guanfacine.	None	Event-related EEG beta activity from midfrontal cortical sources in the ACC differentially predicted improvements in ADHD severity.
Michelson (2007) (97)	ADHD	Examine the effects of CYP2D6 on the efficacy, safety, and tolerability of atomoxetine in children and adolescents from atomoxetine clinical trials.	None	Poor metabolizers had markedly greater reductions in mean symptom severity scores compared with extensive metabolizers.
Nag (2020) (99)	ASD	Test the feasibility of tracking gaze using wearable smart glasses and the ability of these gaze-tracking data to distinguish children with ASD from TD controls.	ML	Wearable smart glasses show promise in identifying subtle differences in gaze tracking and emotion recognition patterns in children with and without ASD, but these differences cannot yet be reliably exploited by ML.
Nakai (2017) (100)	ASD	Compare a ML-based voice analysis with human hearing judgments for classifying children with ASD and TD.	ML	Detected a significant classification difference for identifying children with ASD. The ML-based approach yielded a higher true-positive than false-negative rate, whereas speech therapist judgements yielded similar true-positive and false-negative rates.
Ogrim (2014) (101)	ADHD	Search for predictors of stimulant medication outcomes for ADHD, emphasizing variables from EEG, ERPs^47^, and behavioral data.	None	The clinical outcome of stimulant medication was best predicted by electrophysiological parameters. Responders were primarily a/w prefrontal lobe hypoactivation, whereas non-responders were deviant from the controls in parietal-occipital functions.
Ogrim (2019) (102	ADHD	Predict clinical gains and risks of stimulant medication in pediatric ADHD, combining measures from EEG, ERPs, and behavioral data.	None	Gains and side effects of stimulants in pediatric ADHD can be predicted with high accuracy by combining EEG spectra, ERPs, and behavior from baseline and single-dose tests.
Oruche (2016) (103)	DBD	Explore the feasibility of collecting genetic material from adolescents and their family members and evaluate the association of five single-nucleotide polymorphisms with DBD^48^.	None	Adolescents with DBD had significantly higher minor allele frequencies for SNPs^49^ in DRD2^50^ and DBH^51^ compared to the 1000 Genome Project sample.
Parracho (2005) (104)	ASD	Study the fecal flora of patients with ASD and compare them with those of two control groups.	None	The fecal flora of ASD patients contained a higher incidence of the C. histolyticum group of bacteria than healthy children; however, the non-ASD sibling group had intermediate level of the C. histolyticum group of bacteria.
Pereira-Sanchez (2021) (105)	ADHD	Evaluate the feasibility of conducting a naturalistic neuroimaging study with a clinical sample of children and adolescents with ADHD, with the aim of exploring putative fMRI correlates of differential symptomatic response to stimulant medications.	None	Results showed strengthened negative correlations across pairs of brain regions corresponding to different networks in children with ADHD who responded to lisdexamfetamine after long-term treatment, when contrasted to treatment-naive patients.
Pines (2021) (108)	ASD	Investigate whether individual brain network pathology, either in specific networks or in network engagement characterizes ASD.	None	Despite severe cognitive delays, children with regressive-type ASD may demonstrate intact typical cortical network activation - these intact cognitive networks may not be fully expressed, potentially because aberrant networks interfere with their long-range signaling.
Rádosi (2023) (110)	ADHD	Examine whether associations of fMRI-measured initial response to reward attainment with affectivity and externalizing, internalizing, and alcohol use problems differ between youth at-risk for and not at-risk for ADHD.	None	Neural response to anticipation of reward is differentially a/w ADHD-relevant outcomes depending on ADHD risk - greater superior frontal gyrus response is a/w lower concurrent indices of depressive problems and greater putamen response is a/w greater prospective hazardous alcohol use.
Ran (2022) (111)	MDD	Examine the association between serum extracellular vesicle miRNA^52^ expression and adolescent MDD using high-throughput sequencing and quantitative reverse transcription polymerase chain reaction.	Omics	The combination of mature miRNA and exposure to emotional abuse could diagnose MDD in adolescents with 82.4% sensitivity and 81.6% specificity.
Rijlaarsdam (2021) (112)	GPF	Examine the associations of DNA methylation with general and specific factors of GPF^53^.	Omics	Identified one co-methylated module a/w GPF, with functional characterization of the sites contained in this module suggested that variation may be best explained by environmental rather than genetic influences.
Roberts (2021) (113)	General mental health	Determine the potential utility of PGx^54^ in childhood diseases and identify targets for future pediatric PGx research.	Omics	Most participants had PGx variants that could impact their current treatment, the most prominent of which involved CYP2D6, CYP2C19^55^, and CYP3A5^56^.
Rossi (2011) (114)	ASD	Determine whether children with ASD spectrum disorders with plasma autoantibodies to neural tissue were phenotypically different from autistic children who did not demonstrate anti-brain antibodies.	None	Multiple brain-reactive antibodies in plasma from children with ASD, and TD toddlers, appear to segregate with behavior rather than diagnosis.
Rossow (2020) (115)	MDD	Determine the association between CYP2C19 metabolizer status and risk for escitalopram, citalopram, and sertraline ADEs in children.	None	Sertraline ADEs were more common in normal metabolizers compared to poor or intermediate metabolizers.
Segura (2023) (119)	BP; Schizophrenia	Estimate the age-related epigenetic modifications to assess differences between young individuals at familial high risk and TDs and their a/w environmental stressors.	None	Individuals at high-risk present epigenetic decelerated aging, which is largely in accordance with previous findings.
Sengupta (2008) (151)	ADHD	Examine the association of the COMT Val108/158Met^57^ polymorphism with task-oriented behavior in children with ADHD, and response to MPH treatment.	None	COMT Val108/158Met polymorphism modulates task-oriented behavior, but does not modulate response of task-oriented behavior with MPH treatment.
Stergiakouli (2015) (122)	ADHD	Test whether polygenic risk scores a/w variation in ADHD trait levels in the general population predict ADHD diagnostic status and severity.	None	Increased polygenic score for ADHD traits predicted ADHD status, ADHD severity, and symptom domain.
Suganya (2015) (124	ASD	Explore the use of urine proteomes as objective and reliable biomarkers are crucial for the clinical diagnosis of ASD.	Omics	A total of 118 differential proteins were identified in the urine between autistic and non-autistic children, of which 18 proteins were reported to be related to ASD.
Sun (2018) (125)	ADHD	Identify all cerebral radiomic features related to the diagnosis and subtyping of ADHD to develop classification models for ADHD diagnosis and subtyping.	ML; Omics	Cerebral radiomics-based classification models allowed for the discrimination of patients with ADHD from healthy controls, as well as the separation of the most common ADHD subtypes.
Swatzyna (2017) (126)	ASD	Investigate prevalence of isolated epileptiform discharges in ASD patients, receiving EEGs as part of routine care to guide medication treatment.	None	EEG data identified 36% of participants with isolated epileptiform discharges, with no significant difference between genders.
Thng (2022) (128)	MDD	Evaluate and compare the performance of a vulnerability index, using polygenic risk score, for MDD in young adolescents with sub-clinical symptoms of depression.	None	Depressive symptoms, measured as a continuous variable reported by parents were a/w MDD polygenic risk scores at baseline and follow-up.
Thümmler (2018) (130)	Various mental health disorders	Present PGx results for CYP2D6 genotyping in an inpatient sample of pediatric individuals presenting severe mental illness with repeated psychotropic treatment failure.	Omics	Functional anomalies of CYP2D6 were found in more than half of pediatric inpatients with pharmacoresistant disease
Tini (2022) (131)	Various mental health disorders	Investigate sertraline pharmacokinetics, pharmacodynamics, efficacy, and tolerability across multiple diagnoses.	None	45% concentration due to dose across all diagnoses, but no relationship with response; for separate diagnoses, only OCD response a/w higher dose and concentration.
Vilgis (2018) (134)	MDD	Test longitudinal associations among emotion regulation, PFC function, and MDD severity in adolescent girls.	None	dmPFC^58^ activity at 16 years of age predicted MDD severity at 17 years of age.
Wang (2023) (138)	PTSD	Investigate relationship between PTSD^59^ and polymorphisms of the low-density lipoprotein receptor gene rs5925.	None	Demonstrates that PTSD prevalence in the C allele carriers was higher than that in the TT homozygotes.
West (2014) (140)	ASD	Discover metabolic features present in plasma samples that can discriminate children with ASD from TD children.	ML; Omics	The best performing PLS^60^ model had an accuracy of 81% and a sensitivity of 92%.
Yang (2009) (144)	MDD	Determine if the sub-genial ACC hyperactivity is a/w MDD in adolescents.	None	Depressed adolescents demonstrated greater activation of the subgenual ACC relative to the normal adolescents.
Yang (2016) (142)	ASD	Identify neuroimaging biomarkers to accurately forecast the response to a treatment for ASD.	None	Discovered a brain network in which the pretreatment brain activities predict treatment response to a behavioral intervention.
Yang (2018) (143)	ASD	Identify serum protein markers of ASD.	Omics	Eight potential ASD peptide region biomarkers were identified and validated.
Yap (2010) (145)	ASD	Identify urinary metabolic phenotypes of ASD.	Omics	Changes in gut microbiota metabolism, amino acid metabolism, and nicotinic acid were a/w ASD.
Zhang (2024) (174)	ADHD	Develop a novel hybrid convolutional neural network and long short-term memory model to enable early warning of common mental health risks.	ML	The model achieves an accuracy of 95%, AUC of 97%, precision of 94%, recall of 91%, and F1 score of 92% on held-out test data.
Zhong (2020) (149)	ADHD	Investigate whether the neurodevelopmental genes predict patients’ responses to MPH and ATX^61^.	None	Polygenic risk score significantly predicted symptomatic improvement with ADHD medication.
Non-biological marker studies				
Curry (2006) (46)	MDD	Identify predictors and moderators of response to acute treatments among depressed adolescents based on demographic and clinical parameters.	None	Adolescents who were younger, less chronically depressed, higher functioning, and less hopeless with less suicidal ideation, fewer melancholic features or comorbid diagnoses, and greater expectations for improvement were more likely to benefit acutely.
Edmunds (2022) (48)	ADHD	Examine if comorbid ADHD and anxiety features or EEG measures of engagement moderated the extent to which children benefited from the EF training.	None	EF training improved behavioral inhibition only for children with clinically significant co-occurring ADHD features; meanwhile anxiety features, while prevalent, did not moderate EF training efficacy.
Elahi (2024) (50	ADHD	Identify subgroups of TD adolescents and adolescents with ADHD based on rating scales and behavioral task performance assessing emotion, irritability, impulsivity, risk-taking, future orientation, and processing speed.	None	Identified four classes: 1) High-Complex Challenges; 2) Moderate-Mixed Challenges; 3) Non-Emotive Impulsivity; and 4) High Regulation and Control.
Ford (2023) (54)	Anhedonia; MDD	Investigate symptom network patterns in adolescents from a GBA biopsychosocial perspective. Test the GBA Pathways Systems Theory relationship and investigate symptom networks for their overall associations with anhedonia and depressed mood.	Multimodal profile	The GBA perspective revealed several symptom neighbors that could expand clinical assessment, diagnosing criteria, education, and interventions for adolescents at risk for, or with, anhedonia or depressed mood: weight loss, self-worth tied to weight, difficulty sustaining attention, poor eye contact, etc.
Lavigne (2024) (82	ADHD	Provides information on the likelihood of an ADHD diagnosis early in elementary school for children who have certain symptoms earlier but may not meet diagnostic criteria for ADHD in preschool.	Big data; ML	A classification tree analysis conducted at age 4 predicted age 6 ADHD-any diagnosis 65.82% better than chance; an age 5 CTA^62^ predicted age 6 ADHD-any 70.60% better than chance.
Li (2019) (85)	Pervasive behavioral impairment	Investigate whether Child and Adolescent Functional Assessment Scale, sub-scales, demographic and clinical characteristics contribute to post-treatment functioning.	None	Primary presenting problem, caregiver support, and area of residence were a/w initial level of dysfunction, length of treatment, and the presence of pervasive behavioral impairment among children.
Mandelli (2023) (150	ASD	Use early snapshots of adaptive functioning, using VABS^63^, and unsupervised data-driven discovery methods to uncover highly stable early ASD subtypes that yield information relevant to later prognosis.	Multimodal profile; ML; Big data	Demonstrated that a single snapshot of early adaptive functioning from the VABS can be used to predict robust and reproducible data-driven subtype labels that are informative about differential outcomes in adaptive functioning as well as different developmental trajectories in areas like non-verbal cognitive ability, language and motor behavior.
Meehan (2020) (94)	Psychopathology	Develop and validate individualized risk prediction models for psychopathology.	None	Findings offer proof-of-principle evidence that prediction modeling can be useful in supporting identification of victimized children at greatest risk for psychopathology.
Molavi (2020) (98)	ADHD	Explore cognitive correlates of ADHD subtypes based on the Wechsler Intelligence Scale for Children scores; evaluate if the cognitive profile of each ADHD subtype can predict group membership; and assess the level of self-esteem in each ADHD subtype.	None	Cognitive abilities were negatively correlated with inattentive ADHD subtypes and positively correlated with hyperactive ADHD symptoms; ratings of self-esteem also varied by ADHD subtype.
Pugliese (2024) (109)	ASD	Examine whether there are distinct EF phenotypes within subgroups of ASD individuals and whether these phenotypes relate to differential mental health problems.	None	Demonstrated that ASD youth cluster into three distinct EF profiles, and that these EF groups differed on anxiety, aggression, affect, and inattention symptoms.
Rudolph (2017) (116)	MDD	Identify youth at greatest risk for MDD across the critical developmental transition of adolescence based on cognition-emotion predictors.	None	Compromised cognitive control predicted subsequent depressive symptoms in girls, but not boys, with high trait negative emotionality.
Shih (2016) (120	ASD	Predict the percentage of time spent engaged at exit, rather than response or nonresponse to treatment, to understand youth ASD treatment outcomes.	None	Four ASD subgroups, based on children’s playground engagement scores measured at entry and changes from entry to midpoint, were identified to tailor programming prior to treatment end.
Shirafkan (2020) (121)	ADHD	Evaluates the relationship between MPH dosage with treatment response in ADHD to propose an optimal dose on the basis of the individualized factors of each patient.	None	Clinical severity at baseline, dosage of MPH, and duration of receiving MPH were a/w a two-step procedure to make personalized dosage recommendations.
Storch (2022) (123)	ASD	Examine predictors and moderators of treatment response to personalized and standard CBT^64^ for anxiety in youth with ASD.	None	A more time-intensive, parent-involved, and adapted CBT strategy performed better, especially for older ASD youth and youth with the most complex presentations.
Tariq (2019) (127)	ASD	Use ML classifiers based on videos of Bangladeshi children collected from Dhaka Shishu Children’s Hospital to scale an ASD screening tool to another language and cultural context.	ML	The ML technique achieved 85% accuracy in distinguishing children with ASD from children with other types of developmental delays.
Thomson (1998) (129)	ADHD	Investigate the prediction of treatment response in ADHD using multiple demographic and clinical characteristics.	None	The identified factors are only partially predictive of stimulant responsiveness, with the strength of their relationships only having sub-clinical meaning
Tumlin (2023) (132)	PTSD	Develop method to detect different response categories of children exposed to complex trauma.	None	Detected three classes of response: resilient (majority); unfolding symptoms (fewest); missed symptoms (intermediate).
Wang (2021) (137)	OCD	Investigate race/ethnicity differences in patients with OCD.	None	Asian youth reported later ages of OCD symptom onset, clinical diagnosis, and treatment compared with Caucasian youth.
Washington (2020) (139)	ASD	Evaluate the capability and potential of a crowd of virtual workers to aid in the task of diagnosing ASD.	ML	The best worker responses produce accuracy and variability on par with experts according to prior studies.
White (2015) (141)	ASD	Examine the course of anxiety and long-term stability of reductions in anxiety, in adolescents with ASD who received CBT for anxiety.	None	Reduction in anxiety was maintained during the year following treatment, with greater ASD severity predicting better treatment response.
Zhang (2024) (174)	Suicidal ideation	Investigate the developmental trajectories of sleep disturbance symptoms and examine whether specific trajectories predict suicidal ideation.	None	Underscore the importance of identifying individuals at higher risk of sleep disturbance and providing personalized mental health services.
MH intervention implementation studies				
Aggensteiner (2024) (29)	CD/ODD	Design SCL arousal-bio-feedback training to reduce aggression in CD/ODD.	None	The SCL biofeedback treatment was neither superior nor inferior to the active TAU.
Almirall (2016) (31)	ASD	Compare communication outcomes among three adaptive interventions in children with ASD who are minimally-verbal.	None	The adaptive intervention beginning with a combination of “joint attention, symbolic play, engagement and regulation”, “enhanced milieu teaching”, and “speech-generated device” was estimated as superior.
Beidas (2014) (37)	Anxiety	Extend the probability of treatment benefit method by adding treatment condition as a stratifying variable.	None	Study participants had a 58% probability of ending the treatment phase of the study in the normal range on the Pediatric Anxiety Rating Scale, with variability in the probability values depending on baseline severity and treatment condition.
Blais (2025) (39	General mental health	Evaluate feasibility, acceptance of a care pathway using stepped neuropsychological assessment characterize cognitive function of new outpatients, using these profiles to help providers tailor care to individual patients.	Multimodal profile	Neuropsychology-informed pediatric outpatient care was feasible and well-received.
Blasco-Fontecilla (2019) (40)	General mental health	Evaluate the clinical utility of a mental health drug prescription decision support tool.	Omics	The decision support tool helped to improve the clinical outcome as measured by the Clinical Global Impressions Scale in virtually all children. It also helped to reduce the number of children using polypharmacy, the mean number of drugs per children, and self-reported relevant side effects.
Gewirtz (2019) (58	CD/ODD	Determine whether providing parents with choice of intervention improves outcomes for youths with CD/ODD.	None	Moderation analyses indicated that among parents who selected precision treatment, teacher reports of hyperactivity and inattention were significantly improved.
Hautmann (2023) (63)	General mental health	Develop an algorithm for the prediction of the treatment outcome of behavioral and nondirective parent training and to examine the usefulness of the Personalized Advantage Index in deriving individualized treatment recommendations.	Multimodal profile	Families randomized to their PAI^65^-predicted optimal intervention showed a treatment advantage of d = 0.54, 95% CI [0.17, 0.97]; for ADHD, the advantage was negligible at d = 0.35, 95% CI [–0.01, 0.78].
Huang (2021) (66)	ASD	Demonstrate the clinical feasibility and technical implementation of an evidence-based, fully transparent bioinformatics pipeline for whole genome sequencing in youths with ASD.	Omics	Confirmed a portion of the key variants with Sanger sequencing and provided interpretation with consideration of patients’ clinical symptoms and detailed literature review.
Kuehn (2022) (76)	Suicidal behavior	Illustrate one application of the idiographic approach in the context of STB research focusing on person-specific variability in associations between STBs, coping strategies, and ability to refrain from suicidal action.	Multimodal profile	Individuals who report similar suicidal risk levels likely respond in individualized ways to suicidal urges necessitating personalized assessment and treatment.
Kusuma (2024) (78)	Suicidal behavior	Develop separate models to predict suicide attempts within a cohort at middle and late adolescence.	Big data; ML	The late adolescence models performed better than the mid-adolescence models.
McKay (2020) (93)	General mental health	Explore the thoughts of parents of children with behavioral and conduct problems regarding parenting programs and how they could be personalized.	None	Findings point to the potential of personalized approaches to extend the reach of parenting programs to parents and children who do not currently benefit from such programs.
Peris (2013) (106)	OCD	Examine the feasibility and acceptability of a personalized intervention for pediatric OCD^66^ characterized by certain family profiles.	None	Families receiving standard treatment demonstrated a 40% response rate, but families receiving positive family interactive therapy demonstrated a 70% response rate.
Peris (2017) (107)	OCD	Examine the efficacy of a personalized intervention module designed for cases of OCD characterized by certain family profiles.	None	Personalized treatment demonstrated a clear advantage in terms of overall response and remission rates, and reductions in functional impairment; likewise, personalized treatment outperformed standard treatment on measures of family functioning, producing significantly better reductions in symptom accommodation and family conflict.
Sabatello (2021) (117)	General mental health	Investigate views of teens on translating genetic-based knowledge about psychiatric risks into preventive behaviors.	None	Found high interest among the population in learning about genetic and environmental factors contributing to psychiatric disorders.
VandeVoort (2022) (133	MDD	Investigate effect of psycho-PGx testing in clinical decision making in treatment of MDD.	Omics	No outcome differences between TAU and treatment including PGx.
Voss (2019) (135)	ASD	Evaluate the efficacy of a smart device, driven by artificial intelligence for improving social outcomes of children with ASD.	Digital health data	Children receiving the intervention showed significant improvements in socialization compared with TD controls.
Young (2021) (146)	MDD	Evaluate whether MDD prevention programs can be optimized by matching youths to interventions specific to their psychosocial vulnerabilities.	None	Matched adolescents showed greater decreases in depressive symptoms than mismatched adolescents.
Predictive algorithm studies				
Eni (2020) (51)	ASD	Compare the effectiveness of different ML algorithms for predicting the diagnosis of ASD.	ML	A convolutional neural network yielded the best results.
Kusuma (2024) (78)	Suicidal Behaviour	Use supervised ML to prospectively predict suicide attempts in a nationally representative cohort of Australians at two developmental stages: middle (age 14-15) and late (age 18-19) adolescence. A second aim was to compare the models’ features and predictive performance as this cohort ages.	Big data; ML	The overall best-performing model used random forests in late adolescence, with the late adolescence models generally performing better than the mid-adolescence models.
Lamb (2024) (80)	General mental health	Examine the utility of using neurocognitive data in combination with an ML algorithm to predict client selection of strategies in a virtual environment.	None	Neurocognitive data in may be used to successfully predict client outcome and increase the quality and reliability of artificially intelligent counselors and improve counselors use of client-based analytics in face-to-face and digital counseling environments.
Liu (2016) (87)	ASD	Examine whether face scanning patterns could be used to identify children with ASD using ML.	ML	Manifest the effectiveness and feasibility of applying the ML algorithm based on the face scanning patterns in classifying and predicting ASD.
Saggu (2024) (118)	General mental health	Examine the accuracy and performance of GNN^63^ ML models compared to RNN^64^, baseline conventional ML, and regression models for predicting emergency department revisits.	Big data; ML	The GNN model outperformed both the RNN model and the best performing conventional ML model.
Walsh (2018) (136)	Suicidal behavior	Examine whether an ML approach could produce accurate prediction of adolescent suicide attempts.	ML	Computational models outperformed standard logistic regression in prediction of suicide risk.

GI, gastrointestinal; a/w, associated with; ASD, autism spectrum disorder; SCL, skin conductance level; CD/ODD, conduct disorder/oppositional defiant disorder; TAU, treatment as usual; OXT, oxytocin; MPH, methylphenidate; EEG, electroencephalogram; ADHD, attention deficit hyperactivity disorder; APF, alpha peak frequency; EMG, electromyography; MRI, magnetic resonance imaging; fMRI, functional magnetic resonance imaging; CYP2D6, cytochrome P450 family 2 subfamily D member 6; ATX, ataxia; CpG, 5’—C—phosphate—G—3’; RSFC, resting state functional connectivity; MDD, major depressive disorder; PFC, prefrontal cortex; EF, executive function; NR3C1, nuclear receptor subfamily 3 group C member 1; BD, bipolar disorder; TD, typically developing; ML, machine learning; GBA, gut-brain axis; CYP2C9, cytochrome P450 family 2 subfamily C member 9; ABCB1, adenosine-triphosphate-binding cassette sub-family B member 1; NF, neurofeedback; ERP, event-related potential; PRT, pain reprocessing therapy; SLO, structured laboratory observation; ADE, adverse drug effect; FA, fractional anisotropy; PLE, persistent psychotic like experience; ASQ, ages and stages questionnaire; VNTR, variable number of tandem repeats; DAT1, human dopamine transporter; ACC, anterior cingulate cortex; IQ, intelligence quotient; ABCD, adolescent brain cognitive development; TMS, transcranial magnetic stimulation; GABA, gamma-aminobutyric acid; TNS, trigeminal nerve simulation; pdSNV, potentially damaging single nucleotide variation; pdCNV, potentially damaging copy number variation; ERP, event-related potential; DBD, disruptive behavioral disorder; SNP, single-nucleotide polymorphism; DRD2, dopamine receptor subtype D2; DBH, dopamine beta-hydroxylase; MiRNA, micro ribonucleic acid; GPF, general psychopathology factor; PGx, pharmacogenetics; CYP2C19, cytochrome P450 family 2 subfamily C member 19; CYP3A5, cytochrome P450 family 3 subfamily A member 5; COMT Val108/158Met, catechol-O-methyltransferase met for val at codon 108/158; dmPFC, dorsomedial prefrontal cortex; PTSD, post-traumatic stress disorder; PLS, partial least squares; atomoxetine; CTA, classification tree analysis; VABS, vineland adapting behavior scales; CBT, cognitive behavioral therapy; Personalized Advantage Index; OCD, obsessive-compulsive disorder.

The most common study design category was observational: cohort studies (35% (43/124)) (40–44, 53, 57, 61, 64, 65, 67, 69, 72, 74, 77, 78, 80, 82, 83, 85, 91, 94, 98, 102, 109, 112, 113, 115, 116, 118, 120, 121, 126, 131, 134, 136, 137, 139, 142, 147–150); case-control studies (32% (40/124)) (28, 30, 32–36, 38, 45, 47, 50, 52, 55, 56, 62, 68, 70, 79, 81, 84, 85, 87, 95, 99, 100, 103, 104, 108, 110, 111, 114, 119, 122, 124, 125, 127, 130, 140, 143, 145); cross-sectional studies (5% (6/124)) (49, 54, 90, 105, 117, 132); and case series (3% (4/124)) 39, 66, 76, 130. RCTs were used in 19% (24/124) (29, 31, 37, 46, 48, 58–60, 63, 88, 89, 92, 96, 97, 106, 107, 119, 123, 129, 133, 135, 141, 146, 151) of the studies. Two studies used non-randomized controlled trials (75, 102), and one was a qualitative study (93).

**Figure 4:**
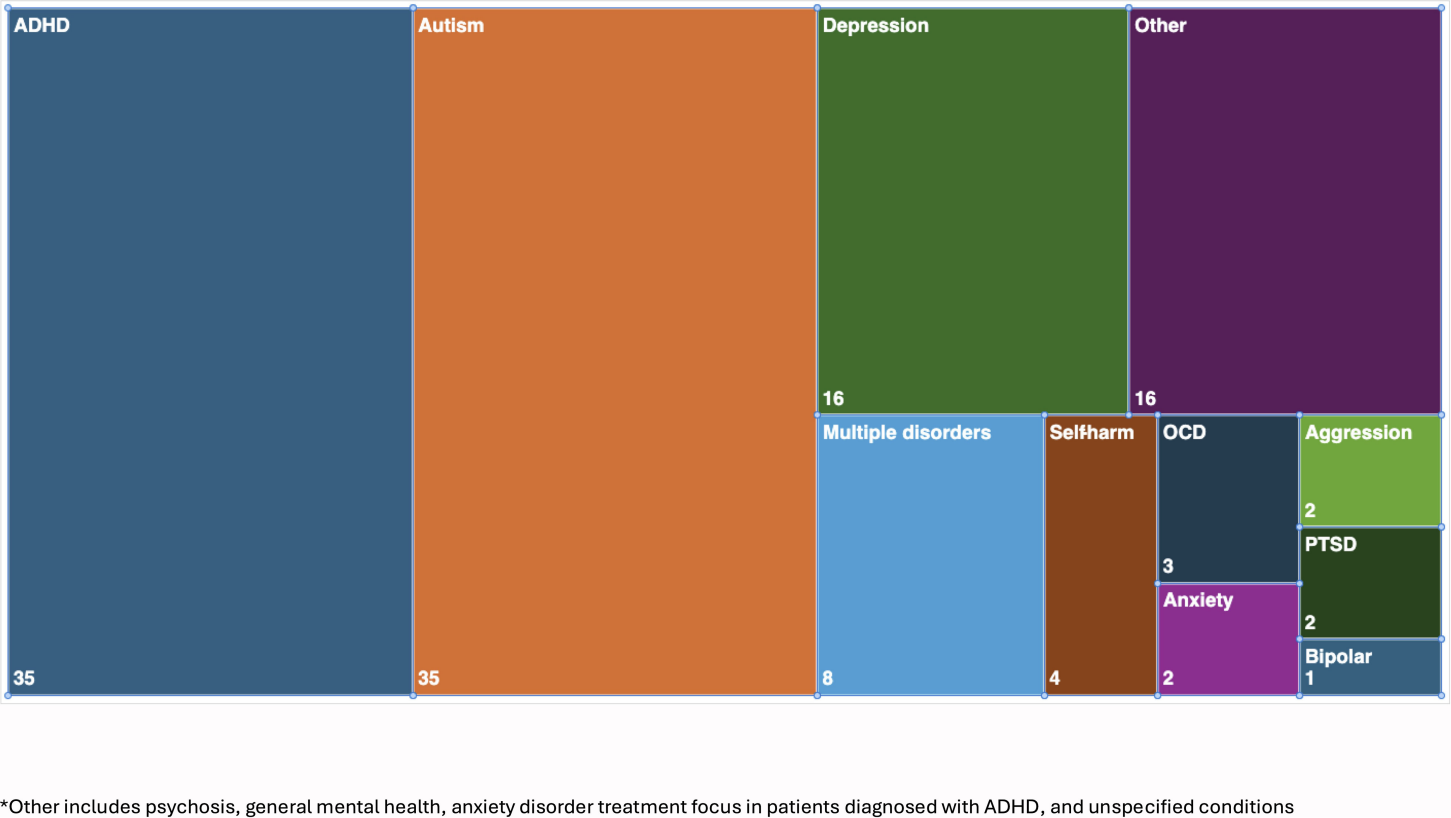
Tree map of clinical foci in studies (n=124).

The clinical focus of a study was defined as either a primary mental health diagnosis, e.g., attention deficit hyperactivity disorder (ADHD) or mental health problem, e.g., suicidality. There were 11 clinical foci, as can be seen in the tree map diagram in **Figure 4**. The most frequent studies were about ADHD (28% (35/124)) (32–34, 36, 41, 42, 44, 50, 59–61, 65, 71–73, 79, 82, 83, 86, 88, 89, 92, 96–98, 101, 102, 105, 110, 121, 122, 125, 129, 149, 151) autism spectrum disorders (ASD) (28% (35/124)) (28, 30, 31, 35, 38, 48, 51, 55, 56, 64, 66, 68, 70, 81, 87, 90, 95, 99, 100, 104, 108, 109, 114, 120, 123, 124, 126, 127, 135, 139, 140, 142–145, 150); and depression (13% (16/124)) (45–47, 53, 54, 74, 77, 84, 111, 115, 116, 128, 133, 134, 144, 146). The fewest studies were on bipolar disorder (52), aggression (62, 75), psychosis (67, 69) and post-traumatic stress disorder (132, 138), each of which only comprised 2% of the study’s set.

A total of 110,386 participants were studied, the count excluding publications which used the same sample more than once. Sample sizes ranged from 6 to 26,055 children and youth, with a mean sample size and standard deviation of 912 ± 3120 and a median of 110 subjects with an interquartile range of 61 to 221. It was found that 12% (15/124) of studies included more than 1,000 participants. In contrast, 10% (12/124) of studies had 20 or fewer participants.

Children were defined as those between the ages of 0 and 10 years, while youth or adolescents were defined as those between the ages of 10 and up to, but not including, 18 years. Samples were further categorized as children only 30% (37/124), youth only 19% (24/124), or mixed 51% (63/124). Girl-only populations were investigated in less than 1% (1/124) (134) of the studies, boy-only populations in 3% (4/124) (33, 36, 79, 86) and mixed-gender populations in 91% (113/124) (28–32, 34–37, 40–45, 47–64, 66–75, 77, 78, 80–85, 87–117, 119–133, 135–146, 148–151). The remaining 6% (7/124) (38, 39, 46, 65, 76, 118, 147) of studies did not adequately describe the gender composition of the populations.

Constructed from the qualitative content analysis were four categories of PCYMH research foci aiming to develop more precise diagnoses, mental health problem definition (e.g., suicidality) prognoses, or prediction of treatment response. Studies collecting data from any biological system with any method were labeled the Biomarker category and comprised (68% (84/124)) of the publications (28, 30, 32–36, 38, 41–45, 47–49, 52–57, 59–62, 64, 65, 67–75, 77, 79, 81, 83, 84, 86, 88–92, 95–97, 99–105, 108, 110–115, 119, 122, 124–126, 128, 130, 131, 134, 138, 140, 142, 143, 145, 147–149, 151).

The Non-Biological Markers category investigated potential markers consisting of specific symptoms of disorders, demographic information, or family history (without genetics data) as predictors in addition to components of the general external exposome, defined by Neufcourt, et al (152) as exposures outside the body, such as social, cultural, and ecological contexts without have specific biological effects. These constituted 17% (22/124) of the papers (46, 48, 50, 54, 82, 85, 94, 98, 109, 116, 120, 121, 123, 127, 129, 132, 137–139, 141, 147, 150).

The third most commonly used PCYMH focus was categorized as Implementation of PCYMH interventions, defined as studies of the feasibility, effectiveness, or acceptability of a novel PCYMH-driven intervention in a clinical setting. These constituted 14% (17/124) of the total dataset (29, 31, 37, 39, 40, 58, 63, 66, 76, 78, 93, 106, 107, 117, 133, 135, 146). The smallest category was Predictive Algorithms (5% (6/124)) (51, 78, 80, 87, 118, 136). Most used machine learning or other AI-assisted modelling to investigate the likelihood that an individual might develop a specific diagnosis, suicidality, or present with repeat emergency department visits.

Categories were not mutually exclusive. Several studies (4% (5/124)) (29, 48, 54, 78, 138) fell into two categories, three of which were in the Biomarker and Non-Biological Marker categories (48, 54, 138), one which was in the Implementation and Biomarker categories (29) and one which was in the Implementation and Predictive Algorithm categories (78). As a result, the percentages presented above do not necessarily add up to 100%.

**Table 3** presents the sub-categories for each of the main categories of PCYMH focus. For instance, of the 84 studies investigating biomarkers, 46% (39/84) (32–36, 38, 43, 45, 47, 48, 53, 54, 59, 60, 64, 68, 69, 71, 72, 74, 75, 79, 83, 84, 86, 88, 89, 96, 101, 102, 105, 108, 110, 125, 126, 134, 142, 144, 148) examined neural biomarkers, 33% (28/84) (30, 41, 42, 44, 49, 57, 65, 67, 71–73, 77, 90, 92, 97, 103, 111–113, 115, 119, 122, 128, 130, 131, 138, 149, 151) investigated genetic biomarkers, and 8% (7/84) (30, 61, 62, 70, 131, 140, 145) investigated metabolite biomarkers. The remaining sub-categories accounted for less than 10% of the studies, including eye tracking, gut biome, peptide, protein, antibody, voice analysis, sleep, circadian rhythm, reaction time and skin conductance biomarkers. Again, the sub-categories were not mutually exclusive, and as such five studies (30, 52, 71, 72, 131) fell into multiple sub-categories.

**Table 3:** Content Analysis: Major types of studies and sub-categories (N = 124).

Categories and sub-categories	n (%)
Biomarkers Neural activity Genetic Metabolite Eye tracking Gut biome Protein Peptide Antibody Skin conductance Sleep Circadian rhythm Reaction time Voice analysis Non-biological markers Phenotype Sociodemographic Behavioral Predictive algorithms Implementation	84 (68) 39 (46) 28 (33) 7 (8) 4 (3) 2 (2) 2 (2) 1 (1) 1 (1) 1 (1) 1 (1) 1 (1) 1 (1) 1 (1) 22 (18) 15 (68) 8 (36) 10 (45) 6 (5) 17 (14)

In contrast, the 22 studies investigating non-biological markers were relatively evenly distributed across the category’s composite sub-categories. Of the studies investigating non-biological markers, 68% (15/22) (46, 54, 82, 85, 94, 98, 109, 116, 121, 123, 129, 132, 137, 141, 150) investigated phenotypic, i.e. symptoms markers, 45% (10/22) (48, 50, 94, 120, 121, 127, 132, 137, 139, 147) investigated behavioral markers, and 36% (8/22) (46, 85, 94, 121, 123, 129, 132, 138) investigated sociodemographic markers. As 36% (8/22) (46, 85, 94, 121, 123, 129, 132, 137) of studies employed multiple types of non-biological markers in tandem, the percentages do not necessarily add up to 100%. Neither the Biomarker nor the Non-Biological Marker categories contained work beyond Phase 1, i.e. identifying a putative marker, in the process of marker development for clinical use (153). A list of PCYMH tools was developed using key foundational literature (19, 26). Of our study set, we found that 19% (23/124) (30, 40, 41, 57, 62, 65, 66, 70–72, 77, 90, 95, 111–113, 124, 125, 130, 133, 140, 143, 145) used -omics methods, 17% (21/124) (51, 52, 59, 61, 68, 72, 78, 79, 82, 87, 91, 99, 100, 118, 125, 127, 136, 139, 140, 148, 150) employed machine learning techniques, 8% (10/124) (39, 54–56, 59, 63, 72, 76, 79, 150) used multimodal profiling, 9% (11/124) (61, 68–70, 72, 77, 78, 82, 118, 148, 150) worked with big data, less than 3% (3/124) (52, 91, 135) employed the use of digital health data and less than1% (1/124) (61) employed virtual populations.

## Discussion

To our knowledge, this is the first scoping review to comprehensively map the PCYMH literature. We retrieved publications using PCYMH and synonymous terms in the title/abstract, advancing understanding of the field’s current scope. Although several position papers and commentaries have outlined the potential of the PCYMH paradigm to improve child and youth mental health (15, 154, 155), the empirical evidence remains limited (156, 157). This is reflected in a relatively small number of eligible studies, the absence of replication, few clinical validation efforts, and a scarcity of implementation-focused PCYMH research. Even the CYMH diagnosis categories with the highest number of articles (ADHD and ASD) only had 1–3 papers about each biomarker or non-biological marker and few implementation studies. Finally, none of the included studies used a reporting guideline, highlighting the lack of standardization across this emerging field.

The recent explosion in publications (49% since 2020) indicates the field is just coming into its own, but the dominance of biomarker studies (67%) compared to the scarcity of implementation studies (14%) tells us that the focus remains on basic discovery rather than clinical application. These features, in combination with only ten studies using individualized multimodal profiles in PCYMH research or care, and the absence of reporting guidelines, leads us to conclude that PCYMH research is in the infancy of its development, with great potential, with many hopes still unrealized.

We know of no other PCYMH reviews with which to compare our results. However, adult precision MH research does appear to be further along. For example, in a systematic review of research through 2019 about precision health or medicine in adults with myriad mental health conditions such as psychotic, mood, anxiety, and substance use disorders, Salazar de Pablo et al. (158) were able to identify 584 prediction modeling studies, estimating individualized risks for diagnosis, prognosis, or treatment response. We suspect that the field of PCYMH may just need more time, as in our dataset, the majority of studies weren’t published until after 2015 (and most in the last five years), when President Obama declared the beginning of the precision medicine era (159).

Another comparator is precision medicine or health research in the non-CYMH pediatric population. Here we also find a more well-developed corpus of work, especially studies identifying molecular targets for treatment in pediatric cancer (160), cardiac disorders (161), and rare diseases (162).

Why isn’t PCYMH research as advanced as precision research in other pediatric disorders? We speculate that PCYMH, like previous research in CYMH diagnosis, treatment, and prevention, is particularly difficult because causation is complex and non-singular (163). Complicating this issue is the transmutation of symptoms and problems within the context of evolving child and youth development (164, 165). Precision health research in non-CYMH pediatric disorders also wrestles with constant change in the individual from normal development, but most pediatric medical disorders have objective markers of pathology, the lack of which has always been a major challenge in CYMH research and care. However, the tools now available in precision health are, for the first time, enabling biological, lifestyle, and environmental discoveries about psychopathology beyond the description of symptoms and behaviours, giving reason for optimism about the future transformational power of PCYMH (154).

In addition, the effects of social determinants of health, including exposure to adverse events, makes the development of PCYMH more complicated (166). It is now accepted that these determinants derail normal child and adolescent development and have consequences well into adulthood, but their effects on CYMH problems are particularly severe and consequential for an individual’s entire life (167). Furthermore, correlates or possible etiologic factors such as poverty, abuse, neglect, or out of home placement are complicated to “treat” and not under the control of any one societal system, including health care.

Precision mental health or psychiatry has been touted as the next scientific revolution (154, 168), but potential barriers to widespread operationalization and uptake are significant, including possible psychological harm to patients, unknown economic consequences, potential increases in mental healthcare disparities, failure to deliver on promises of increased treatment success, and inadequately trained clinical staff, with poor integration of research into care (169).

However, if PCYMH is in its infancy, this is the ideal time to shape this body of research to facilitate maximal opportunity for success. First, standardization of how PCYMH research is planned, conducted, and reported would enhance the quality of studies and enhance inter-study comparison, allowing better of results from multiple studies. The first reporting guideline was published in 1996 (170) and this line of work has steadily expanded in scope and impact. Using such guidelines could have obviated publication of the papers we found with inadequate information on samples and methods.

Second, while developing studies, it is always important to consider and mitigate biases. However, in the context of PCYMH, where the main objective is typically to identify sub-groups needing different diagnosis, treatments or prevention interventions than the “the average” research participant or patient, issues such as selection bias run the risk of significantly undermining the validity of any observations made. Studies using AI, e.g., various types of machine learning to make relevant clinical predictions, are particularly vulnerable to myriad biases (171). Since these studies have the potential to have a large impact on care, identifying and mitigating AI-related bias is particularly important. Evidence-based medicine has significantly advanced CYMH research and it remains relevant and essential for translating PCYMH research findings into real-world impact for children and youth (17). AI-generated algorithms, biomarker, and non-biomarker studies all need to be replicated and validated and a large proportion of this work requires evidence-based medicine methods. Very few studies in our dataset have progressed beyond identification of markers or early development of Ai generated predictive algorithms and this dearth of such studies will undermine advancement of PCYMH (172). Furthermore, validation of algorithms, biomarkers, and non-biological markers by clinicians and persons with lived experience who represent the population of interest, whether a clinical condition or the community, is critical to close the standard multi-year gap between research discoveries and impact. PCYMH will not succeed if results do not penetrate clinical care settings or the community.

Evaluating the implementation and outcomes of PCYMH interventions—both in care and prevention—is essential. There was only one study that did this for a PCYMH care intervention feasibility study (39). If there were more such studies, the knowledge mobilization rate would increase and sharing of ideas and data could significantly advance the field.

Our data show that PCYMH tools are not yet being used to full capacity. Some studies aimed to answer PCYMH questions, but they used no PCYMH tools at all, relying on more traditional designs or data analytic methods. Neither were most studies yet at the stage of using multiple types of data, e.g. neuroimaging, genetics, wearable digital health cardiovascular function information, and lifestyle characteristics to develop the individualized multimodal profiles crucial to administering tailored mental health care. The five studies (29, 48, 54, 78, 138) falling into two categories of types of PCYMH research may be harbingers of future research in which studies will routinely characterize individuals using multiple types of data.

A goal of our review was to determine if there were any diagnostic or MH problem groups for which there was adequate PCYMH research to conduct a systematic review or meta-analysis. Unfortunately, even for ASD and ADHD, the two disorders with the largest number of articles, there too few papers about any specific PCYMH sub-category or tool to proceed to more comprehensive types of reviews.

### Strengths and limitations

This scoping review has several strengths. Its broad, but structured guiding question and the comprehensive analysis of studies provide a valuable “snapshot” of the current PCYMH research landscape. By mapping the existing literature, the review offers an overview that can inform future, more focused inquiries.

There are also three limitations. First, restricting inclusion to English-language publications introduces the potential for language bias, with possible exclusion of useful non-English papers. Second, using two databases, PubMed and Embase, while appropriate for a scoping review, may have resulted in missing relevant publications indexed in other databases, e.g., PsycInfo. In addition, exclusion of the grey literature, a decision made because it would not answer our core question about published literature, means that some emerging research or practice-based knowledge may not have been discovered. Future reviews, whether scoping or systematic, should ensure interrogation of these other sources.

Finally, as with all scoping reviews, the intent was to map and describe rather than to evaluate the quality or strength of evidence. This approach is valuable for the first high-level analysis of a scoping review, but limits the certainty with which conclusions can be drawn.

## Conclusions

This review shows that PCYMH research, while still in its infancy, has made rapid progress between 2020 and 2024. Among all the publications, there were four types of PCYMH research: (1) Biomarker; (2) Non-Biological Marker; (3) Implementation of PCYMH Interventions: and (4) Predictive Algorithms studies. This corpus of research investigated eleven CYMH diagnoses or problems, the latter most prominently represented by studies on suicidality and self-harm. The current state of knowledge and implementation of the PCYMH paradigm is primed for improvement in the depth and breadth of studies, sharpening the focus to fill gaps in the discovery process, and improving knowledge mobilization.

### Recommendations for future PCYMH research

We recommend the following changes in PCYMH research to advance the paradigm from early development to practice.

1. The clinical foci of this body of work were dominated by studies of ASD and ADHD. While important CYMH diagnoses, we recommend that the clinical scope of PCYMH research be expanded to include anxiety and mood disorders. Like ASD and ADHD, these clinical designations identify heterogeneous groups of children and youth, which could be improved with the PCYMH paradigm. Expanding the body of PCYMH work to include these two could have a tremendous impact on the CYMH population, as these disorders affect 20-25% of 3-17-year-olds worldwide (173, 174).
2. We found gaps in the specific types of PCYMH research. For example, of the four phases of biomarker development (identification, verification, evaluation against a gold standard, and testing in a clinical setting) (153), the PCYMH biomarker research primarily is in the early phases. We recommend that researchers continue to advance their work further along the well-described phases of validation and clinical care pathway developments, with large enough samples to detect sub-group-based differences informing PCYMH usage.
3. PCYMH research about Non-Biological Markers could benefit from using a similar structure as biomarker development (recommendation #2), where phases instead capture functional, social, or psychological outcomes rather than biological ones.
4. Implementation of PCYMH intervention studies should be driven by implementation science methodology. While many healthcare-based, implementation science frameworks do not directly address precision medicine, Mogagka and colleagues (175) synthesized the four most commonly used frameworks and aligned the constructs with the tenets of precision medicine to create a precision medicine implementation framework. Future PCYMH researchers may find this helpful.
5. The Predictive Algorithms PCYMH focus category also provides opportunities for improvement, especially since we expect increased growth in AI in this category. None of the studies we found reported an AI bias analysis in the project design, which can be of considerable concern with AI research. We recommend structured analysis of possible biases, accompanied by prevention or mitigation strategies while developing study plans for PCYMH research (176, 177) or when carrying out prediction studies (171).
6. Plan PCYMH programs of research that can produce multimodal profiles of individuals, i.e., biological and non-biological predictors for diagnosis, treatment response, prognosis, or prevention.
7. Given that privacy and ethics concerns (178), as well as clinician skepticism (169) abound in PCYMH research, we recommend research teams include clinicians and persons with lived experience from the design stage throughout a study to optimize research uptake and validity.
8. Use reporting guidelines in study planning and to increase knowledge mobilization of PCYMH research findings. Examples of such reporting guidelines are: the Transparent Reporting of a multivariable prediction model for Individual Prognosis Or Diagnosis (TRIPOD) (179), the Better Precision-data Reporting of Evidence from Clinical Intervention Studies & Epidemiology (BePRECISE) (180), and the Generative Artificial intelligence tools in MEdical Research (GAMER) (181).
9. Incorporate evaluation of implementation outcomes in all PCYMH care or prevention studies and ensure widespread knowledge mobilization through publication and conference presentation of these evaluations.

## Data Availability

The original contributions presented in the study are included in the article/**Supplementary Material**. Further inquiries can be directed to the corresponding author/s.
