# Supplementary material for: Scoping review of precision child and youth mental health research: dwelling in possibility
Source: Front Psychiatry. 2026 Feb 9;16:1691548. doi: 10.3389/fpsyt.2025.1691548 (PMC12926772; doi:10.3389/fpsyt.2025.1691548)
Supplement: Supplementary file 2 [file Table2.docx]

**Supplementary Table 2. PubMed search strategy**

| **Concept** | **Keywords** | **Results** |
| --- | --- | --- |
| **Precision health:** tailoring a health intervention taking into account a patient’s specific biological, lifestyle, and/or environmental characteristics. | “individualized behavioral health” [Title/Abstract] OR “individualized health” [Title/Abstract]OR “individualized medicine” [Title/Abstract]OR “individualized mental health” [Title/Abstract]OR “individualized psychiatry” [Title/Abstract]OR “personalized behavioral health” [Title/Abstract]OR “personalized health” [Title/Abstract]OR “personalized medicine” [Title/Abstract]OR “personalized mental health” [Title/Abstract]OR “personalized psychiatry” [Title/Abstract]OR “precision behavioral health” [Title/Abstract]OR “precision health” [Title/Abstract]OR “precision medicine” [Title/Abstract]OR “precision mental health” [Title/Abstract]OR “precision psychiatry” [Title/Abstract]OR “Precision Medicine” [MeSH Terms] | 62,253 |
| **Youth:** patients between 0 to 18 years of age. | adolescen* [Title/Abstract] OR child [Title/Abstract]OR children [Title/Abstract]OR teen* [Title/Abstract]OR youth* [Title/Abstract]OR "Adolescent" [MeSH Terms] OR “Child” [MeSH Terms] | 3,888,043 |
| **Mental health:** all topics related to the origin, prevention, diagnosis, and treatment of mental health conditions. | “behavioral health” [All Fields] OR “mental health” [All Fields] OR psychiatr* [All Fields] OR "Mental Health" [MeSH Terms] | 1,243,217 |
| **Precision + youth + mental health** | (“individualized behavioral health” [Title/Abstract]OR “individualized health” [Title/Abstract]OR “individualized medicine” [Title/Abstract]OR “individualized mental health” [Title/Abstract]OR “individualized psychiatry” [Title/Abstract]OR “personalized behavioral health” [Title/Abstract]OR “personalized health” [Title/Abstract]OR “personalized medicine” [Title/Abstract]OR “personalized mental health” [Title/Abstract]OR “personalized psychiatry” [Title/Abstract]OR “precision behavioral health” [Title/Abstract]OR “precision health” [Title/Abstract]OR “precision medicine” [Title/Abstract]OR “precision mental health” [Title/Abstract]OR “precision psychiatry” [Title/Abstract]OR “Precision Medicine” [MeSH Terms]) AND (adolescen* [Title/Abstract]OR child [Title/Abstract]OR children [Title/Abstract]OR teen* [Title/Abstract]OR youth* [Title/Abstract]OR "Adolescent" [MeSH Terms] OR “Child” [MeSH Terms]) AND (“behavioral health” [All Fields] OR “mental health” [All Fields] OR psychiatr* [All Fields] OR "Mental Health" [MeSH Terms]) | 422 |
