# Supplementary material for: Scoping review of precision child and youth mental health research: dwelling in possibility
Source: Front Psychiatry. 2026 Feb 9;16:1691548. doi: 10.3389/fpsyt.2025.1691548 (PMC12926772; doi:10.3389/fpsyt.2025.1691548)
Supplement: Supplementary file 3 [file Table3.docx]

**Supplementary Table 3. Embase search strategy**

| **Concept** | **Keywords** | **Results** |
| --- | --- | --- |
| **Precision health:**tailoring a health intervention taking into account a patient’s specific biological, lifestyle, and/or environmental characteristics. | exp personalized medicine/ OR (individualized behavioral health OR individualized health OR individualized medicine OR individualized mental health OR individualized psychiatry OR personalized behavioral health OR personalized health OR personalized medicine OR personalized mental health OR personalized psychiatry OR precision behavioral health OR precision health OR precision medicine OR precision mental health OR precision psychiatry).ti,ab | 93,709 |
| **Youth:** patients between 0 to 18 years of age. | exp adolescent/ OR exp child/ OR exp juvenile/ OR (adolescen* OR child OR children OR teen* OR youth*).ti,ab. | 5,049,699 |
| **Mental health:** all topics related to the origin, prevention, diagnosis, and treatment of mental health conditions. | exp child psychiatry/ OR exp mental health/ OR psychiatry/ OR (behavioral health OR mental health* OR psychiatr*).ti,ab. | 796,275 |
| **Precision + youth + mental health** | (exp personalized medicine/ OR (individualized behavioral health OR individualized health OR individualized medicine OR individualized mental health OR individualized psychiatry OR personalized behavioral health OR personalized health OR personalized medicine OR personalized mental health OR personalized psychiatry OR precision behavioral health OR precision health OR precision medicine OR precision mental health OR precision psychiatry).ti,ab) AND (exp adolescent/ OR exp child/ OR exp juvenile/ OR (adolescen* OR child OR children OR teen* OR youth*).ti,ab.) AND (exp child psychiatry/ OR exp mental health/ OR psychiatry/ OR (behavioral health OR mental health* OR psychiatr*).ti,ab.) | 458 |
