# Supplementary material for: Scoping review of precision child and youth mental health research: dwelling in possibility
Source: Front Psychiatry. 2026 Feb 9;16:1691548. doi: 10.3389/fpsyt.2025.1691548 (PMC12926772; doi:10.3389/fpsyt.2025.1691548)
Supplement: Supplementary file 4 [file Table4.docx]

**Supplementary Table 4. Details of biomarker studies by first author’s last name (n=84)**

| **First Author (Publication Year)** | **Country** | **Aim** | **PCYMH Tools** | **Design** | **Secondary Analysis** | **Sample** | **Key Findings** |
| --- | --- | --- | --- | --- | --- | --- | --- |
| Adams (2011) | USA | Identify gut flora and GI^1^ biomarkers a/w^2^ ASD^3^. | None | Case-control | Y | N = 97 (58 youth with ASD; 39 controls)  29.9% female  2.5-18 years | GI symptoms were strongly correlated with the severity of ASD. |
| Aggensteiner (2024) | Germany | Design SCL^4^ arousal-bio-feedback training to reduce aggression in CD/ODD^5^. | None | RCT^6^ | Y | N = 37 (youth with CD/ODD)  5.4% female  8-14 years | The SCL biofeedback treatment was neither superior nor inferior to the active TAU^7^. |
| Al-Ali (2022) | Iraq | Assess the clinical utility of blood OXT^8^ serum levels and receptor genotype as biomarkers of ASD. | Omics | Case-control | N | N = 120 (60 youth with ASD; 60 controls)  25.0% female  3-15 years | Peripheral OXT levels and OXT receptor genetic alterations were identified as potential biomarkers of social functioning in the ASD patient setting. |
| Arnett (2022) | USA | Measure the association between positive response to MPH^9^ treatment and abnormal frontal-striatal neural network excitation. | None | Case-control | Y | N = 49 (29 youth with ADHD^10^; 50 controls)  30.5% female  7-11 years | MPH responders demonstrated attenuated P3 amplitude relative to controls, while responders did not differ on this measure. |
| Arns (2008) | Netherlands | Investigate the predictive value of EEG^11^ phenotypes for stimulant medication effectiveness in ADHD. | None | Case-control | N | N = 98 (49 youth with ADHD; 49 controls)  0% female  6-18 years | The Frontal Slow and Slowed APF^12^ and the Low Voltage EEG phenotype discriminated ADHD subjects best from controls. |
| Arns (2018) | Netherlands | Replicate and extend EEG biomarkers used to predict non-response to ADHD medication. | None | Case-control | Y | N = 494 (336 youth with ADHD; 158 controls)  27.7% female  Ages not stated | For treatment prediction, clear gender and age-group differences were found, where a low APF in male adolescents with ADHD was a/w a smaller likelihood of responding to MPH. |
| Baker (2021) | USA | Measure association between social motivation and neural changes from pre- to post-intervention in ASD. | None | Case-control | N | N = 27 (13 youth with ASD; 14 controls)  18.5% female  11-18 years | Parent-reported social responsiveness and social skills improved in adolescents with ASD after participation in a precision medicine intervention. |
| Bazanova (2018) | Russia | Determine EEG and EMG^13^ biomarkers most related to the main ADHD characteristics. | None | Case-control | N | N = 117 (94 youth with ADHD; 23 controls)  0% female  Ages not stated | ADHD children were characterized with decreased individual APF, alpha bandwidth and alpha amplitude suppression magnitude, as well as with increased alpha1/alpha2 ratio and scalp muscle tension. |
| Bernas (2017) | Netherlands | Propose a novel MRI^14^-based ASD biomarker by analyzing temporal brain dynamics in resting-state fMRI^15^. | None | Case-control | N | N = 54 (24 youth with ASD; 30 controls)  Sex not stated  Age range not stated | Study shows change in the coherence of temporal neurodynamics is a biomarker of ASD, and wavelet coherence-based classifiers lead to robust and replicable results which could be used as an objective ASD diagnostic tool. |
| Brown (2016) | USA | Characterize the effect of CYP2D6^16^ genotype on the dose-exposure relationship for atomoxetine. | Omics | Cohort | N | N = 23 (youth with ADHD)  13.0% female  9.5-17.8 years | Dose-corrected ATX^17^ systemic exposure varied 29.6-fold across the study cohort. Simulated steady state profiles at the maximum US Food and Drug Administration-recommended dose suggest that most patients are unlikely to attain adequate ATX exposures. |
| Bruxel (2013) | Brazil | Evaluate the association between a 75 T4G polymorphism and appetite reduction as a side effect of MPH in children with ADHD. | None | Cohort | N | N = 205 (youth with ADHD)  26.3% female  4-17 years | The G allele presented a trend for association with appetite reduction scores; however, the G allele carriers presented a higher risk for appetite reduction worsening when compared with T allele homozygotes. |
| Cardinale (2023) | USA | Disentangle cognitive control correlates of anxiety versus irritability. | None | Cohort | Y | N = 89 (youth)  59.55% female  2-15 years | Results of whole-brain multivariate linear models revealed that anxiety at age 15 was uniquely a/w decreased neural response to conflict across multiple regions implicated in attentional control and conflict adaptation. |
| Carpentieri (2023) | Italy | Search for clinical biomarkers of ADHD in CpG^18^ methylation patterns. | None | Cohort | N | N = 19 (youth with ADHD)  15.8% female  6-12 years | For “improving” ADHD children, CpGs 3 and 5 were methylated with CpG 2 and CpG 6; however, for “severe” ADHD children, CpGs 2 and 5 accompanied a methylated CpG 1. |
| Connolly (2017) | USA | Examine whether amygdala RSFC^19^ is a/w changes in MDD^20^ severity in adolescents. | None | Case-Control | N | N = 101 (48 youth with MDD; 53 controls)  61.4% female  Age range not stated | Compared to healthy controls, depressed adolescents showed reduced amygdala-based RSFC with the dorsolateral PFC^21^ and the ventromedial PFC. |
| Doruk Camsari (2019) | USA | Examine baseline measures of cortical inhibition and excitability in depressed patients and characterize their longitudinal posttreatment changes. | None | Case-control | Y | N = 37 (15 youth with MDD; 22 controls)  54.1% female  9-17 years | Baseline, short-interval intracortical inhibition-2 was significantly reduced in depressed participants, suggesting impaired cortical inhibition compared with healthy controls. |
| Edmunds (2022) | USA | Examine if comorbid ADHD and anxiety features or EEG measures of engagement moderated the extent to which children benefitted from the EF^22^ training. | None | RCT | Y | N = 70 (youth with ASD)  10.0% female  7-11 years | EF training improved behavioral inhibition only for children with clinically significant co-occurring ADHD features; meanwhile anxiety features, while prevalent, did not moderate EF training efficacy. |
| Efstathopoulos (2018) | Sweden | Examine the association between NR3C1^23^ methylation and the emergence of internalizing symptoms in childhood and adolescence. | None | Cross-sectional | Y | N = 1149 (youth)  54.4% female  13-14 years | NR3C1 hypermethylation was cross-sectionally a/w high score for internalizing symptoms in the whole group as well as among the female participants. |
| Faedda (2016) | USA | Test the hypothesis that objective measures of activity, sleep, and circadian rhythms would help differentiate pediatric subjects with BD^24^ from ADHD and TD^25^ controls. | ML^26^; Digital Health Data | Case-control | N | N = 155 (48 youth with BD; 44 youth with ADHD; 21 youth with ADHD and comorbid MDD; 42 controls)  37.4% female  5-18 years | There were prominent group differences in several activity measures, notably mean 5 lowest hours of activity, skewness of diurnal activity, relative circadian amplitude, and vulnerability index. |
| Forbes (2010) | USA | Provide preliminary evidence that pretreatment reward-related brain function in the striatum and medial PFC could have relevance for predicting both final level and rate of change of clinical characteristics in adolescents with MDD. | None | Cohort | N | 13 youth with MDD)  69.2% female  10-16 years | Final levels of severity and anxiety symptoms were a/w pretreatment striatal reactivity, and rate of anxiety symptom reduction was a/w greater striatal reactivity and lower medial PFC reactivity. |
| Ford (2023) | USA | Investigate symptom network patterns in adolescents from a GBA^27^ biopsychosocial perspective. Test the GBA Pathways Systems Theory relationship and investigate symptom networks for their overall associations with anhedonia and depressed mood. | Multimodal profile | Cross-sectional | Y | N = 11,607 (youth)  47.8% female  9-11 years | The GBA perspective revealed several symptom neighbors that could expand clinical assessment, diagnosing criteria, education, and interventions for adolescents at risk for, or with, anhedonia or depressed mood: weight loss, self-worth tied to weight, difficulty sustaining attention, poor eye contact, etc. |
| Frazier (2016) | USA | Create an objective, eye tracking-based ASD risk index. | Multimodal profile | Case-control | N | N1 = 45 (25 youth with ASD; 20 TD)  32.33% female  3-8.11 years  N2 = 24 (15 youth with ASD; 19 TD)  17.17% female  3-8.11 years | In both samples, the ASD risk index had high diagnostic accuracy, was strongly a/w Autism Diagnostic Observation Schedule–Second Edition severity scores, and not significantly correlated with language ability. |
| Frazier (2018) | USA | Develop and validate eye tracking-based measures for estimating ASD risk and quantifying ASD symptom levels. | Multimodal profile | Case-control | N | N = 201 (youth with ASD)  19.9% female  1.6-17.6 years | Eye tracking measures appear to be useful quantitative, objective measures of ASD risk and ASD symptom levels. |
| Gassó (2014) | Spain | Evaluate the influence of CYP2D6, CYP2C9^28^ and ABCB1^29^ genotypes on the steady-state plasma concentrations of fluoxetine and its active metabolite (S)-norfluoxetine, and on the clinical improvement in patients receiving fluoxetine treatment. | Omics | Cohort | N | N = 83 (youth with MDD, OCD^30^, or anxiety)  68.7% female  10-17 years | Results confirm the influence of CYP2D6 genetic variants in fluoxetine pharmacokinetics and provide evidence for the potential effect of the ABCB1 genotype on the clinical improvement in children and adolescent patients treated with fluoxetine. |
| Ging-Jehil (2023) | USA | Explore whether baseline cognitive processing moderated the effect of NF^31^ on improvement in the composite parent- and teacher-rated inattention score from baseline to end-treatment. | ML; Multimodal profile | RCT | Y | N = 133 (youth with ADHD)  22.6% female  7-10 years | Pre-treatment cognitive testing with computational modeling identified children who benefitted more from NF than control treatment for ADHD. |
| Griffiths (2019) | Australia | Explore differences in ERPs^32^ that are a/w noradrenergic activity—N2 and P3—in atomoxetine responders versus non-responders. | None | RCT | Y | N = 104 (52 youth with ADHD; 52 controls)  19.2% female  6-17 years | Responders were distinguished by significantly lower auditory oddball N2 amplitudes than both non-responders and TD controls, particularly in the right frontocentral region. |
| Gutiérrez-Casares (2021) | Spain | Model the efficacies of the two drugs, lisdexamfetamine and MPH, and compare them in a virtual head-to-head setting. Describe an approach to measure and compare the output results in terms of efficacy of the two medications, the molecular mechanisms triggered, and the response to ADHD management in a diverse population of virtual patients, including patients with the most common psychiatric comorbidities. | Big data; ML; Virtual population | Cohort | Y | N = 500 (youth)  20.0% female  6-17 years | Provided adult and pediatric-adolescent virtual populations and generated quantitative systems pharmacology models to infer the mechanism of action of lisdexamfetamine and MPH. |
| Hagenbeek (2020) | Netherlands | Examine the association of urinary metabolites and neurotransmitter ratios involved in key metabolic and neurotransmitter pathways in a cohort of twins and clinic-referred children, using biomarker panels to identify correlates of aggression. | Omics | Case-control | Y | N = 1,530 (youth with aggression)  45.3% female  3-10 years | 6 biomarkers were significantly a/w childhood aggression, of which the association of O-phosphoserine, and gamma-L-glutamyl-L-alanine remained significant after multiple testing. |
| Hegarty (2019) | USA | Examine the relationships between structural measures of language regions in the brain and changes in language abilities following PRT^33^ in young children with ASD. | None | Cohort | Y | N = 18 (youth with ASD)  16.7% female  2-6 years | Level of improvement on the SLO^34^ was correlated with baseline asymmetry of the inferior frontal gyri, while the size of the left superior temporal gyri at baseline was correlated with the level of improvement on standardized parental questionnaires. |
| Hong (2012) | South Korea | Investigate the interactions between dopamine transporter gene, dopamine D4 receptor gene, alpha-2A adrenergic receptor gene, and norepinephrine transporter gene in ADHD treatment response to MPH. | Omics | Cohort | N | N = 103 (youth with ADHD)  Sex not stated  Age range not stated | Genetic determinants of MPH response consist of both dopaminergic and noradrenergic gene polymorphisms. |
| Ivashchenko (2020) | Russia | Analyze possible associations of gene polymorphisms with the effectiveness and safety of antipsychotics in adolescents with an acute psychotic episode for 14 days of treatment. | None | Cohort | N | N = 53 (youth with an acute psychotic episode)  47.2% female  10-18 years | Established that CYP2D6 intermediate metabolizer phenotype and polymorphisms ABCB1 2677G>T/A and 3435C>T were significantly a/w a higher frequency of several ADEs^35^. |
| Jiang (2023) | China | Identify and model brain-wide differences in structural connectivity using diffusion tensor imaging in young ASD and TD children. | Big data, ML | Case-control | Y | N = 119 (93 youth with ASD; 26 controls)  11.8% female  4-6 years | Revealed the presence of a small number of inter-regional structural connections within the brains of young children with ASD which exhibit increased FA^36^ compared to TD and negatively a/w symptom severity. |
| Karcher (2023) | USA | Examine the degree to which persistent and distressing PLEs^37^ exhibit neural metrics that show similarity to adults with chronic psychiatric and neurologic conditions. | Big data | Cohort | Y | N = 8,242 (youth with persistent and distressing psychotic-like experiences)  47.5% female  9-10 years | Findings suggest that especially the persistent distressing PLEs in children were a/w neural metrics resembling those observed in adults with severe psychiatric and neurologic conditions. |
| Kelly (2019) | USA | Investigate blood and stool metabolomic profiles a/w the ASQ^38^ derived communication score, as a proxy for ASD risk, in children from the Vitamin D Antenatal Asthma Reduction Trial, a clinical trial of prenatal vitamin D supplementation and outcomes in pregnant women and their offspring. | Big data; Omics | Case-control | Y | N = 403 (38 youth with low ASQ scores; 365 controls)  46.8% female  3-8 years | Identified a number of metabolomic pathways and metabolites with biologically plausible relationships with impaired development of communication skills and with ASD risk. |
| Kim (2011) | South Korea | Evaluate the effects of val66met on hippocampal volume and on encoding-related hippocampal activity while considering the potential influence of childhood abuse and diagnostic status. | Omics | Cohort | N | N = 102 (youth with ADHD)  19.6% female  6-12 years | Val66met was found to have a significant impact on hippocampal volume independently of childhood abuse and psychiatric status. |
| Kim (2015) | South Korea | Examine whether applying ML to pretreatment demographic, clinical, environmental, neuropsychological, neuroimaging, and genetic information can predict ADHD therapeutic response following MPH administration. | ML; Omics; Big data; Multimodal profile | Cohort | N | N = 83 (youth with ADHD)  21.7% female  Age range not stated | Findings support an association between homozygosity for the Val allele and better response to MPH in Korean ADHD children as assessed by four different response criteria. |
| Kirley (2003) | Ireland | Examine a dopamine transporter gene as conferring susceptibility to ADHD. | None | Cohort | Y | N = 119 (youth with ADHD)  5.9% female  Age range not stated | There is an association between the 10-repeat VNTR^39^ DAT1^40^ polymorphism and retrospectively rated MPH response. |
| Klimes-Dougan (2022) | USA | Evaluate if baseline structure and function of the amygdala and ACC^41^ predict response to Interpersonal Psychotherapy for Depressed Adolescents. | None | Cohort | Y | N = 15 (youth with MDD)  80.0% female  12-16 years | The following were a/w greater improvement to MDD symptoms: greater ACC activation during an emotion-matching task and greater amygdala-ACC resting-state functional connectivity. There was minimal evidence that brain structure predicted changes in depressive symptoms. |
| Klimes-Dougan (2018) | USA | Examine the stress activation and response system to: differentiate high versus low-risk children, and to explore indicators a/w favorable intervention response. | None | Non-RCT | N | N = 43 (youth with elevated aggression or socially withdrawn behavior)  41.9% female  Age range not stated | Findings provide preliminary evidence that hypothalamic pituitary adrenal axis biological variables may be helpful tools for identifying children who would benefit from intervention and personalizing interventions. |
| Kurkinen (2023) | Finland | Discover metabolic alterations in sexually or physically abused depressed adolescent psychiatric outpatients. | Big data; Omics | Cohort | Y | N = 76 (youth with MDD)  84.2% female  14-18 years | Revealed alterations in metabolites related to one-carbon metabolism, mitochondrial dysfunction, oxidative stress, and inflammation in depressed patients with a history of sexual or physical abuse. |
| Kyeong (2017) | South Korea | Determine whether new ADHD clinical phenotypes can be identified based on symptom severity and IQ^42^ measurements. A second aim was to investigate whether neuroimaging findings validate identified phenotypes. | ML; Multimodal profile | Case-control | Y | N = 263 (158 youth with ADHD; 105 controls)  0% female  No age range stated | Demonstrated that the use of common clinical phenotypes and an innovative unsupervised data-driven ML algorithm is an informative approach for understanding the heterogeneity of ADHD. |
| Latrèche (2021) | Switzerland | Examine the relationship between social orienting and baseline clinical characteristics in young children with ASD, as well as explored the role of social orienting as a predictor of developmental change and treatment outcome. | None | Case-control | Y | N = 111 (95 youth with ASD; 16 controls)  16.2% female  1.39-3.96 years | Attention to face is robustly correlated with ASD symptoms, developmental skills, developmental change, and verbal gains in particular. Social orienting predicted a better treatment outcome in the context of an early and intensive intervention, paving the way toward. |
| Lee (2022) | USA | Use the ABCD^43^ dataset to examine shared and non-shared neural correlates of response inhibition and error processing across distinct phenotypes of ADHD, irritability, and their co-occurrence using data-driven, latent variable modeling techniques. | None | Cohort | Y | N = 5,948 (youth with ADHD)  52.9% female  9-10 years | Latent class analysis revealed four phenotypic groups based on severity of ADHD and co-occurring irritability. Group differences emerged in the neural coactivation network a/w response inhibition but not error processing. |
| Lewis (2016) | USA | Examine the relationship between MDD severity and TMS^44^ measures of cortical inhibition and excitability in children and adolescents | None | Case-control | N | N = 46 (24 youth with MDD; 22 controls)  54.3% female  9-17 years | Preliminary results provide evidence for a relationship between MDD severity and dysfunction in GABA^45^ergic and glutamatergic cortical processes. |
| Lim (2013) | UK | Apply Gaussian process classification to grey matter volumetric data to assess whether ADHD adolescents can be accurately differentiated from healthy controls based on objective, brain structure measures. | None | Case-control | N | N = 87 (29 youth with ADHD; 19 youth with ASD; 29 controls)  0% female  10.7-17.9 years | Discriminating grey matter patterns showed higher association between ADHD and earlier developing ventrolateral/premotor fronto-temporo-limbic. |
| Loo (2016) | USA | Compare effects of MPH, guanfacine, and combination of the two on resting state EEG and determine if these a/w improvements in behavioral and cognitive functioning. | None | RCT | Y | 179 youth with ADHD  30.7% female  7-14 years | Revealed distinct underlying medication-related effects on neural mechanisms. The combination condition uniquely exhibited an EEG profile that was a/w improved behavioral and cognitive functioning. |
| Loo (2021) | USA | Test cognitive and EEG predictors of treatment response with ADHD. | None | RCT | N | N = 51 (youth with ADHD)  33.3% female  8-12 years | Those with more severe executive dysfunction are more likely to be TNS^46^ responders, show modulation of right frontal brain activity, improved/normalized EFs, and ADHD symptom reduction. |
| Mahjani (2021) | USA | Evaluate the frequency of pdSNVs^47^ and their impact on medical and psychiatric phenotypes relative to pdCNVs^48^. | Omics | Cross-sectional | Y | 996 (youth with ASD)  29.2% female  Age range not stated | Rare pdSNVs were more common than pdCNVs, with the combined yield of potentially damaging variation was substantial at 27%. The results provide compelling rationale for the use of high-throughput sequencing as part of routine clinical workup for ASD. |
| McGinnis (2019) | USA | Use ML to identify children with internalizing disorders using an instrumented 90-second fear induction task. | Digital health data; ML | Cohort | Y | N = 63 (youth with various mental illnesses)  57.1% female  3-8 years | When paired with ML, the data collected from 20 seconds of wearable sensor use during a fear induction task can be used to identify young children with internalizing disorders with a high level of accuracy, sensitivity, and specificity. |
| McGough (2006) | USA | Explore genetic moderators of symptom reduction and side effects in MPH-treated children with ADHD. | None | RCT | Y | N =165 (youth with ADHD)  26.1% female  3-5.5 years | Emerging evidence suggests the potential for optimizing ADHD medications on the basis of individual genetics. |
| Meng (2021) | China | Identify differential proteins in the urinary proteome between ASD and non-ASD children aged 3–7 years. | Omics | Case-control | N | N = 24 (18 youth with ASD; 6 youth with TD)  12.5% female  3-8 years | 118 differential proteins were identified in the urine between ASD and non-ASD children, with cadherin-related family member 5 and vacuolar protein sorting-associated protein 4B showing the best discriminative ability. |
| Michelini (2023) | USA | Investigate pretreatment clinical and EEG profiles as predictors of treatment outcome in children randomized to MPH and guanfacine. | None | RCT | Y | N = 181 (youth with ADHD)  32.0% female  7-14 years | Event-related EEG beta activity from midfrontal cortical sources in the ACC differentially predicted improvements in ADHD severity. |
| Michelson (2007) | USA | Examine the effects of CYP2D6 on the efficacy, safety, and tolerability of atomoxetine in children and adolescents from atomoxetine clinical trials. | None | RCT | Y | N = 3,861 (youth with ADHD)  22.2% female  6-18 years | Poor metabolizers had markedly greater reductions in mean symptom severity scores compared with extensive metabolizers. |
| Nag (2020) | USA | Test the feasibility of tracking gaze using wearable smart glasses and the ability of these gaze-tracking data to distinguish children with ASD from TD controls. | ML | Case-control | N | N = 33 (16 youth with ASD; 17 controls)  33.3% female  6-17 years | Wearable smart glasses show promise in identifying subtle differences in gaze tracking and emotion recognition patterns in children with and without ASD, but these differences cannot yet be reliably exploited by ML. |
| Nakai (2017) | Japan | Compare a ML-based voice analysis with human hearing judgments for classifying children with ASD and TD. | ML | Case-control | N | N = 81 (30 youth with ASD; 51 controls)  35.8% female  3-10 years | Detected a significant classification difference for identifying children with ASD. The ML-based approach yielded a higher true-positive than false-negative rate, whereas speech therapist judgements yielded similar true-positive and false-negative rates. |
| Ogrim (2014) | Norway | Search for predictors of stimulant medication outcomes for ADHD, emphasizing variables from EEG, ERPs^49^, and behavioral data. | None | Non-RCT | Y | N = 188 (98 youth with ADHD; 90 controls)  33.3% female  7-17 years | The clinical outcome of stimulant medication was best predicted by electrophysiological parameters. Responders were primarily a/w prefrontal lobe hypoactivation, whereas non-responders were deviant from the controls in parietal-occipital functions. |
| Ogrim (2018) | Norway | Predict clinical gains and risks of stimulant medication in pediatric ADHD, combining measures from EEG, ERPs, and behavioral data. | None | Cohort | N | N = 87 (youth with ADHD)  31.0% female  8-17 years | Gains and side effects of stimulants in pediatric ADHD can be predicted with high accuracy by combining EEG spectra, ERPs, and behavior from baseline and single-dose tests. |
| Oruche (2016) | USA | Explore the feasibility of collecting genetic material from adolescents and their family members and evaluate the association of five single-nucleotide polymorphisms with DBD^50^. | None | Case-control | Y | N = 31 (15 youth with DBD; 16 family members)  33.3% female  Age range not stated | Adolescents with DBD had significantly higher minor allele frequencies for SNPs^51^ in DRD2^52^ and DBH^53^ compared to the 1000 Genome Project sample. |
| Parracho (2005) | UK | Study the fecal flora of patients with ASD and compare them with those of two control groups. | None | Case-control | N | N = 80 (58 youth with ASD; 10 TD unrelated controls; 12 TD sibling controls)  23.8% female  2-16 years | The fecal flora of ASD patients contained a higher incidence of the C. histolyticum group of bacteria than healthy children; however, the non-ASD sibling group had intermediate level of the C. histolyticum group of bacteria. |
| Pereira-Sanchez (2021) | Spain | Evaluate the feasibility of conducting a naturalistic neuroimaging study with a clinical sample of children and adolescents with ADHD, with the aim of exploring putative fMRI correlates of differential symptomatic response to stimulant medications. | None | Cross-sectional | Y | N = 56 (youth with ADHD)  37.5% female  7-17 years | Results showed strengthened negative correlations across pairs of brain regions corresponding to different networks in children with ADHD who responded to lisdexamfetamine after long-term treatment, when contrasted to treatment-naive patients. |
| Pines (2021) | USA | Investigate whether individual brain network pathology, either in specific networks or in network engagement characterizes ASD. | None | Case-control | N | N = 10 (5 youth with ASD; 5 youth controls with epilepsy)  40.0% female  4-16 years | Despite severe cognitive delays, children with regressive-type ASD may demonstrate intact typical cortical network activation - these intact cognitive networks may not be fully expressed, potentially because aberrant networks interfere with their long-range signaling. |
| Rádosi (2023) | Hungary | Examine whether associations of fMRI-measured initial response to reward attainment with affectivity and externalizing, internalizing, and alcohol use problems differ between youth at-risk for and not at-risk for ADHD. | None | Case-control | Y | N = 129 (50 youth with ADHD; 79 TD controls)  38.0% female  14-17 years | Neural response to anticipation of reward is differentially a/w ADHD-relevant outcomes depending on ADHD risk - greater superior frontal gyrus response is a/w lower concurrent indices of depressive problems and greater putamen response is a/w greater prospective hazardous alcohol use. |
| Ran (2022) | China | Examine the association between serum extracellular vesicle miRNA^58^ expression and adolescent MDD using high-throughput sequencing and quantitative reverse transcription polymerase chain reaction. | Omics | Case-control | N | N = 89 (43 youth with MDD; 46 controls)  70.8% female  13-18 years | The combination of mature miRNA and exposure to emotional abuse could diagnose MDD in adolescents with 82.4% sensitivity and 81.6% specificity. |
| Rijlaarsdam (2021) | Netherlands | Examine the associations of DNA methylation with general and specific factors of GPF^55^. | Omics | Cohort | Y | N = 440 (youth with GPF)  49.3% female  10 years | Identified one co-methylated module a/w GPF, with functional characterization of the sites contained in this module suggested that variation may be best explained by environmental rather than genetic influences. |
| Roberts (2021) | USA | Determine the potential utility of PGx^56^ in childhood diseases and identify targets for future pediatric PGx research. | Omics | Cohort | N | N = 452 (youth)  48.2% female  Age range not stated | Most participants had PGx variants that could impact their current treatment, the most prominent of which involved CYP2D6, CYP2C19^57^, and CYP3A5^58^. |
| Rossi (2011) | USA | Determine whether children with ASD spectrum disorders with plasma autoantibodies to neural tissue were phenotypically different from autistic children who did not demonstrate anti-brain antibodies. | None | Case-control | Y | N = 129 (86 youth with ASD; 43 controls)  22.5% female  2-6 years | Multiple brain-reactive antibodies in plasma from children with ASD, and TD toddlers, appear to segregate with behavior rather than diagnosis. |
| Rossow (2020) | USA | Determine the association between CYP2C19 metabolizer status and risk for escitalopram, citalopram, and sertraline ADEs in children. | None | Cohort | N | N = 249 (youth with antidepressant use)  61.8% female  0-18 years | Sertraline ADEs were more common in normal metabolizers compared to poor or intermediate metabolizers. |
| Segura (2023) | Spain | Estimate the age-related epigenetic modifications to assess differences between young individuals at familial high risk and TDs and their a/w environmental stressors. | None | Case-control | Y | N = 117 (53 youth at high risk for schizophrenia and BD; 64 controls)  54.7% female  6-17 years | Individuals at high-risk present epigenetic decelerated aging, which is largely in accordance with previous findings. |
| Sengupta (2008) | Canada | Examine the association of the COMT Val108/158Met^59^ polymorphism with task-oriented behavior in children with ADHD, and response to MPH treatment. | None | RCT | N | N = 188 (youth with ADHD)  15.4% female  6-12 years | COMT Val108/158Met polymorphism modulates task-oriented behavior, but does not modulate response of task-oriented behavior with MPH treatment. |
| Stergiakouli (2015) | UK | Test whether polygenic risk scores a/w variation in ADHD trait levels in the general population predict ADHD diagnostic status and severity. | None | Case-control | Y | N = 20,570 (14,062 youth in discovery sample; 508 youth with ADHD; 6,000 TD)  50.3% female  5-17 years | Increased polygenic score for ADHD traits predicted ADHD status, ADHD severity, and symptom domain. |
| Suganya (2021) | India | Explore the use of urine proteomes as objective and reliable biomarkers are crucial for the clinical diagnosis of ASD. | Omics | Case-control | N | N = 24 (18 youth with ASD; 6 youth with TD)  12.5% female  3-8 years | A total of 118 differential proteins were identified in the urine between autistic and non-autistic children, of which 18 proteins were reported to be related to ASD. |
| Sun (2018) | China | Identify all cerebral radiomic features related to the diagnosis and subtyping of ADHD to develop classification models for ADHD diagnosis and subtyping. | ML; Omics | Case-control | N | N = 170 (40 youth with ADHD inattentive subtype; 43 youth with ADHD combined subtype; 87 TD)  15.9% female  7-15 years | Cerebral radiomics-based classification models allowed for the discrimination of patients with ADHD from healthy controls, as well as the separation of the most common ADHD subtypes. |
| Swatzyna (2017) | USA | Investigate prevalence of isolated epileptiform discharges in ASD patients, receiving EEGs as part of routine care to guide medication treatment. | None | Cohort | N | N = 124 (non-epileptic youth with ASD)  22.6% female  4-18 years | EEG data identified 36% of participants with isolated epileptiform discharges, with no significant difference between genders. |
| Thng (2022) | UK | Evaluate and compare the performance of a vulnerability index, using polygenic risk score, for MDD in young adolescents with sub-clinical symptoms of depression. | None | Cohort | Y | N = 3,825 (youth with sub-clinal depression)  47.0% female  9-13 years | Depressive symptoms, measured as a continuous variable reported by parents were a/w MDD polygenic risk scores at baseline and follow-up. |
| Thümmler (2018) | France | Present PGx results for CYP2D6 genotyping in an inpatient sample of pediatric individuals presenting severe mental illness with repeated psychotropic treatment failure. | Omics | Case series | N | N = 9 (youth)  44.4% female  11-16 years | Functional anomalies of CYP2D6 were found in more than half of pediatric inpatients with pharmacoresistant disease |
| Tini (2022) | Germany | Investigate sertraline pharmacokinetics, pharmacodynamics, efficacy, and tolerability across multiple diagnoses. | None | Cohort | Y | N = 78 (youth with various mental health diagnoses treated with sertraline)  62.8% female  7-18 years | 45% concentration due to dose across all diagnoses, but no relationship with response; for separate diagnoses, only OCD response a/w higher dose and concentration. |
| Vilgis (2018) | USA | Test longitudinal associations among emotion regulation, PFC function, and MDD severity in adolescent girls. | None | Cohort | Y | N = 78 (youth)  100% female  16-17 years | dmPFC^60^ activity at 16 years of age predicted MDD severity at 17 years of age. |
| Wang (2023) | China | Investigate relationship between PTSD^61^ and polymorphisms of the low-density lipoprotein receptor gene rs5925. | None | Cohort | N | N = 709 (youth with and without PTSD)  56.0% female  Age range not stated | Demonstrates that PTSD prevalence in the C allele carriers was higher than that in the TT homozygotes. |
| West (2014) | USA | Discover metabolic features present in plasma samples that can discriminate children with ASD from TD children. | ML; Omics | Case-control | N | N = 82 (52 youth with ASD; 30 controls)  18.3% female  4-6 years | The best performing PLS^62^ model had an accuracy of 81% and a sensitivity of 92%. |
| Yang (2009) | USA | Determine if the sub-genial ACC hyperactivity is a/w MDD in adolescents. | None | Case-control | N | N = 26 (13 youth with MDD; 13 controls)  53.8% female  13-17 years | Depressed adolescents demonstrated greater activation of the subgenual ACC relative to the normal adolescents. |
| Yang (2016) | USA | Identify neuroimaging biomarkers to accurately forecast the response to a treatment for ASD. | None | Cohort | N | N = 20 (youth with ASD)  35.0% female  Age range not stated | Discovered a brain network in which the pretreatment brain activities predict treatment response to a behavioral intervention. |
| Yang (2018) | China | Identify serum protein markers of ASD. | Omics | Case-control | N | N = 148 (68 youth with ASD; 80 controls)  21.6% female  2.6-7 years | Eight potential ASD peptide region biomarkers were identified and validated. |
| Yap (2010) | UK | Identify urinary metabolic phenotypes of ASD. | Omics | Case-control | N | N = 101 (39 youth with ASD; 62 controls)  34.7% female  3-9 years | Changes in gut microbiota metabolism, amino acid metabolism, and nicotinic acid were a/w ASD. |
|  |  |  |  |  |  |  |  |
| Zhang (2024) | China | Develop a novel hybrid convolutional neural network and long short-term memory model to enable early warning of common mental health risks. | ML | Cohort | N | N = 79 (35 youth with ADHD; 44 controls)  Sex not stated  Age range not stated | The model achieves an accuracy of 95%, AUC of 97%, precision of 94%, recall of 91%, and F1 score of 92% on held-out test data. |
| Zhong (2020) | China | Investigate whether the neurodevelopmental genes predict patients’ responses to MPH and ATX^63^. | None | Cohort | N | N = 241 (youth with ADHD)  16.2% female  Age range not stated | Polygenic risk score significantly predicted symptomatic improvement with ADHD medication. |
| 1. GI = gastrointestinal   2. a/w = associated with   3. ASD = autism spectrum disorder   4. SCL = skin conductance level   5. CD/ODD = conduct disorder / oppositional defiant disorder  6. RCT = randomized control trial  7. TAU = treatment as usual   8. OXT = oxytocin   9. MPH = methylphenidate  10. ADHD = attention deficit hyperactivity disorder  11. EEG = electroencephalogram  12. APF = alpha peak frequency  13. EMG = electromyography  14. MRI = magnetic resonance imaging  15. fMRI = functional magnetic resonance imaging  16. CYP2D6 = cytochrome P450 family 2 subfamily D member 6  17. ATX = ataxia  18. CpG = 5'—C—phosphate—G—3'  19. RSFC = resting state functional connectivity  20. MDD = major depressive disorder  21. PFC = prefrontal cortex  22. EF = executive function  23. NR3C1 = nuclear receptor subfamily 3 group C member 1  24. BD = bipolar disorder  25. TD = typically developing  26. ML = machine learning  27. GBA = gut-brain axis  28. CYP2C9 = cytochrome P450 family 2 subfamily C member 9  29. ABCB1 = adenosine-triphosphate-binding cassette sub-family B member 1  30. OCD = obsessive-compulsive disorder  31. NF = neurofeedback  32. ERP = event-related potential  33. PRT = pain reprocessing therapy  34. SLO = structured laboratory observation  35. ADE = adverse drug effect  36. FA = fractional anisotropy  37. PLE = persistent psychotic like experience  38. ASQ = ages and stages questionnaire  39. VNTR = variable number of tandem repeats  40. DAT1 = human dopamine transporter  41. ACC = anterior cingulate cortex  42. IQ = intelligence quotient  43. ABCD = adolescent brain cognitive development  44. TMS = transcranial magnetic stimulation  45. GABA = gamma-aminobutyric acid  46. TNS = trigeminal nerve simulation  47. pdSNV = potentially damaging single nucleotide variation  48. pdCNV = potentially damaging copy number variation  49. ERP = event-related potential  50. DBD = disruptive behavioral disorder  51. SNP = single-nucleotide polymorphism  52. DRD2 = dopamine receptor subtype D2  53. DBH = dopamine beta-hydroxylase  54. MiRNA = micro ribonucleic acid  55. GPF = general psychopathology factor  56. PGx = pharmacogenetics  57. CYP2C19 = cytochrome P450 family 2 subfamily C member 19  58. CYP3A5 = cytochrome P450 family 3 subfamily A member 5  59. COMT Val108/158Met = catechol-O-methyltransferase met for val at codon 108/158  60. dmPFC = dorsomedial prefrontal cortex  61. PTSD = post-traumatic stress disorder  62. PLS = partial least squares  63. ATX = atomoxetine | | | | | | | |
