# Supplementary material for: Scoping review of precision child and youth mental health research: dwelling in possibility
Source: Front Psychiatry. 2026 Feb 9;16:1691548. doi: 10.3389/fpsyt.2025.1691548 (PMC12926772; doi:10.3389/fpsyt.2025.1691548)
Supplement: Supplementary file 5 [file Table5.docx]

**Supplementary Table 5. Details of non-biological marker studies by first author’s last name (N=22)**

| **First Author (Publication Year)** | **Country** | **Aim** | **PCYMH Tools** | **Design** | **Secondary Analysis** | **Sample** | **Key Findings** |
| --- | --- | --- | --- | --- | --- | --- | --- |
| Curry (2006) | USA | Identify predictors and 100% moderators of response to acute treatments among depressed adolescents based on demographic and clinical parameters. | None | RCT^1^ | Y | N = 439 (youth with MDD^2^)  Sex not stated  Age range not stated | Adolescents who were younger, less chronically depressed, higher functioning, and less hopeless with less suicidal ideation, fewer melancholic features or comorbid diagnoses, and greater expectations for improvement were more likely to benefit acutely. |
| Edmunds (2022) | USA | Examine if comorbid ADHD^3^ and anxiety features or EEG^4^ measures of engagement moderated the extent to which children benefited from the EF^5^ training. | None | RCT | Y | N = 70 (youth with ASD^6^)  10.0% female  7-11 years | EF training improved behavioral inhibition only for children with clinically significant co-occurring ADHD features; meanwhile anxiety features, while prevalent, did not moderate EF training efficacy. |
| Elahi (2024) | USA | Identify subgroups of TD^7^ adolescents and adolescents with ADHD based on rating scales and behavioral task performance assessing emotion, irritability, impulsivity, risk-taking, future orientation, and processing speed. | None | Case-control | Y | N = 152 (83 youth with ADHD; 6 youth with subclinical ADHD; 63 TD)  34.9% female  12-18 years | Identified four classes: 1) High-Complex Challenges; 2) Moderate-Mixed Challenges; 3) Non-Emotive Impulsivity; and 4) High Regulation and Control. |
| Ford (2023) | USA | Investigate symptom network patterns in adolescents from a GBA^8^ biopsychosocial perspective. Test the GBA Pathways Systems Theory relationship and investigate symptom networks for their overall associations with anhedonia and depressed mood. | Multimodal profile | Cross-sectional | Y | N = 11,607 (youth)  47.8% female  9-11 years | The GBA perspective revealed several symptom neighbors that could expand clinical assessment, diagnosing criteria, education, and interventions for adolescents at risk for, or with, anhedonia or depressed mood: weight loss, self-worth tied to weight, difficulty sustaining attention, poor eye contact, etc. |
| Lavigne (2023) | USA | Provides information on the likelihood of an ADHD diagnosis early in elementary school for children who have certain symptoms earlier but may not meet diagnostic criteria for ADHD in preschool. | Big data; ML^9^ | Cohort | Y | N = 796 (91 youth with ADHD; 685 controls)  49.1% female  4-6 years | A classification tree analysis conducted at age 4 predicted age 6 ADHD-any diagnosis 65.82% better than chance; an age 5 CTA^10^ predicted age 6 ADHD-any 70.60% better than chance. |
| Li (2019) | Canada | Investigate whether Child and Adolescent Functional Assessment Scale, sub-scales, demographic and clinical characteristics contribute to post-treatment functioning. | None | Cohort | N | N = 1,327 (youth)  48.2% female  6-17 years | Primary presenting problem, caregiver support, and area of residence were a/w^11^ initial level of dysfunction, length of treatment, and the presence of pervasive behavioral impairment among children. |
| Mandelli (2022) | USA | Use early snapshots of adaptive functioning, using VABS^12^, and unsupervised data-driven discovery methods to uncover highly stable early ASD subtypes that yield information relevant to later prognosis. | Multimodal profile; Machine Learning; Big Data | Cohort | Y | 1,812 (1,216 youth with ASD; 689 controls)  27.5% female  0.5-6 years | Demonstrated that a single snapshot of early adaptive functioning from the VABS can be used to predict robust and reproducible data-driven subtype labels that are informative about differential outcomes in adaptive functioning as well as different developmental trajectories in areas like non-verbal cognitive ability, language and motor behavior. |
| Meehan (2020) | UK | Develop and validate individualized risk prediction models for psychopathology. | None | Cohort | Y | N = 591 (youths with history of childhood victimization)  49.9% female  5-18 years | Findings offer proof-of-principle evidence that prediction modeling can be useful in supporting identification of victimized children at greatest risk for psychopathology. |
| Molavi (2020) | Iran | Explore cognitive correlates of ADHD subtypes based on the Wechsler Intelligence Scale for Children scores; evaluate if the cognitive profile of each ADHD subtype can predict group membership; and assess the level of self-esteem in each ADHD subtype. | None | Cohort | N | N = 139 (youth with ADHD)  19.4% female  Age range not stated | Cognitive abilities were negatively correlated with inattentive ADHD subtypes and positively correlated with hyperactive ADHD symptoms; ratings of self-esteem also varied by ADHD subtype. |
| Pugliese (2024) | USA | Examine whether there are distinct EF phenotypes within subgroups of ASD individuals and whether these phenotypes relate to differential mental health problems. | None | Cohort | N | N = 397 youth with ASD  21.4% female  8-14 years | Demonstrated that ASD youth cluster into three distinct EF profiles, and that these EF groups differed on anxiety, aggression, affect, and inattention symptoms. |
| Rudolph (2017) | USA | Identify youth at greatest risk for MDD across the critical developmental transition of adolescence based on cognition-emotion predictors. | None | Cohort | N | N = 636 (youth)  53.1% female  Age range not stated | Compromised cognitive control predicted subsequent depressive symptoms in girls, but not boys, with high trait negative emotionality. |
| Shih (2014) | USA | Predict the percentage of time spent engaged at exit, rather than response or nonresponse to treatment, to understand youth ASD treatment outcomes. | None | Cohort | Y | N = 92 (youth with ASD)  18.5% female  Age range not stated | Four ASD subgroups, based on children’s playground engagement scores measured at entry and changes from entry to midpoint, were identified to tailor programming prior to treatment end. |
| Shirafkan (2020) | Iran | Evaluates the relationship between MPH^13^ dosage with treatment response in ADHD to propose an optimal dose on the basis of the individualized factors of each patient. | None | Cohort | N | N = 221 (youth with ADHD)  23.5% female  3-13 years | Clinical severity at baseline, dosage of MPH, and duration of receiving MPH were a/w a two-step procedure to make personalized dosage recommendations. |
| Storch (2022) | USA | Examine predictors and moderators of treatment response to personalized and standard CBT^14^ for anxiety in youth with ASD. | None | RCT | N | N = 167 (youth with ASD and elevated anxiety)  20.4% female  7-13 years | A more time-intensive, parent-involved, and adapted CBT strategy performed better, especially for older ASD youth and youth with the most complex presentations. |
| Tariq (2019) | Bangladesh | Use ML classifiers based on videos of Bangladeshi children collected from Dhaka Shishu Children’s Hospital to scale an ASD screening tool to another language and cultural context. | ML | Case-control | N | N = 150 (50 youth with ASD; 50 youth with other speech or language conditions; 50 controls)  40.0% female  1.5-4 years | The ML technique achieved 85% accuracy in distinguishing children with ASD from children with other types of developmental delays. |
| Thomson (1998) | Norway | Investigate the prediction of treatment response in ADHD using multiple demographic and clinical characteristics. | None | RCT | N | N = 336 (youth with ADHD)  17.3% female  3-16 years | The identified factors are only partially predictive of stimulant responsiveness, with the strength of their relationships only having sub-clinical meaning |
| Tumlin (2023) | USA | Develop method to detect different response categories of children exposed to complex trauma. | None | Cross-sectional | N | N = 15,883 (youth)  47.9% female  0-18 years | Detected three classes of response: resilient (majority); unfolding symptoms (fewest); missed symptoms (intermediate). |
| Wang (2021) | Canada | Investigate race/ethnicity differences in patients with OCD^15^. | None | Cohort | N | N = 218 (youth with OCD)  78.9% female  Age range not stated | Asian youth reported later ages of OCD symptom onset, clinical diagnosis, and treatment compared with Caucasian youth. |
| Wang (2023) | China | Investigate the relationship between PTSD^16^ and polymorphisms of the low-density lipoprotein receptor gene rs5925. | None | Cohort | N | N = 709 (youth with and without PTSD)  56.0% female  Age range not stated | Demonstrates that PTSD prevalence in the C allele carriers was higher than that in the TT homozygotes. |
| Washington (2020) | USA | Evaluate the capability and potential of a crowd of virtual workers to aid in the task of diagnosing ASD. | ML | Cohort | N | N = 24 (youth with ASD)  50.0% female  Age range not stated | The best worker responses produce accuracy and variability on par with experts according to prior studies. |
| White (2015) | USA | Examine the course of anxiety and long-term stability of reductions in anxiety, in adolescents with ASD who received CBT for anxiety. | None | RCT | Y | N = 22 (youth with ASD and anxiety disorder)  27.3% female  12-17 years | Reduction in anxiety was maintained during the year following treatment, with greater ASD severity predicting better treatment response. |
| Zhang (2024) | China | Investigate the developmental trajectories of sleep disturbance symptoms and examine whether specific trajectories predict suicidal ideation. | None | Cohort | N | N = 19,905 (youth)  48.8% female  10-18 years | Underscore the importance of identifying individuals at higher risk of sleep disturbance and providing personalized mental health services. |
| 1. RCT = randomized control trial  2. MDD = major depressive disorder  3. ADHD = attention deficit hyperactivity disorder  4. EEG = electroencephalogram  5. EF = executive function  6. ASD = autism spectrum disorder  7. TD = typically developing  8. GBA = gut-brain axis  9. ML = machine learning  10. CTA = classification tree analysis  11. a/w = associated with  12. VABS = vineland adapting behavior scales  13. MPH = methylphenidate  14. CBT = cognitive behavioral therapy  15. OCD = obsessive-compulsive disorder  16. PTSD = post-traumatic stress disorder | | | | | | | |
