# Supplementary material for: Scoping review of precision child and youth mental health research: dwelling in possibility
Source: Front Psychiatry. 2026 Feb 9;16:1691548. doi: 10.3389/fpsyt.2025.1691548 (PMC12926772; doi:10.3389/fpsyt.2025.1691548)
Supplement: Supplementary file 6 [file Table6.docx]

**Supplementary Table 6. Details of PCYMH implementation studies by first author’s last name (N=17)**

| **First Author (Publication Year)** | **Country** | **Aim** | **PCYMH Tools** | **Design** | **Secondary Analysis** | **Sample** | **Key Findings** |
| --- | --- | --- | --- | --- | --- | --- | --- |
| Aggensteiner (2024) | Germany | Design SCL^1^ arousal-bio-feedback training to reduce aggression in CD/ODD^2^. | None | RCT^3^ | Y | N = 37 (youth with CD/ODD)  5.4% female  8-14 years | The SCL biofeedback treatment was neither superior nor inferior to the active TAU^4^. |
| Almirall (2016) | USA | Compare communication outcomes among three adaptive interventions in children with ASD^5^ who are minimally verbal. | None | RCT | Y | N = 61 (youth with ASD)  16.9% female  5-8 years | The adaptive intervention beginning with a combination of “joint attention, symbolic play, engagement and regulation”, “enhanced milieu teaching”, and “speech-generated device” was estimated as superior. |
| Beidas (2014) | USA | Extend the probability of treatment benefit method by adding treatment condition as a stratifying variable. | None | RCT | Y | N = 488 (youth with anxiety)  49.6% female  7-17 years | Study participants had a 58% probability of ending the treatment phase of the study in the normal range on the Pediatric Anxiety Rating Scale, with variability in the probability values depending on baseline severity and treatment condition. |
| Blais (2025) | Canada | Evaluate feasibility, acceptance of a care pathway using stepped neuropsychological assessment to characterize cognitive function of new outpatients, using these profiles to help providers tailor care to individual patients. | Multimodal profile | Case series | N | N = 30 (eligible youth patients)  Sex not stated  8-17 years | Neuropsychol-ogy- informed pediatric outpatient care was feasible and well-received. |
| Blasco-Fontecilla  (2019) | Spain | Evaluate the clinical utility of a mental health drug prescription decision support tool. | Omics | Cohort | N | N = 20 (youth with ADHD^6^, ASD, or MDD^7^ – 50% in foster care)  55.0% female  0-17 years | The decision support tool helped to improve the clinical outcome as measured by the Clinical Global Impressions Scale in virtually all children. It also helped to reduce the number of children using polypharmacy, the mean number of drugs per children, and self-reported relevant side effects. |
| Gewirtz (2018) | USA | Determine whether providing parents with a choice of intervention improves outcomes for youths with CD/ODD. | None | RCT | N | N = 129 (youth with CD/ODD)  34.9% female  5-12 years | Moderation analyses indicated that among parents who selected precision treatment, teacher reports of hyperactivity and inattention were significantly improved. |
| Hautmann (2023) | Germany | Develop an algorithm for the prediction of the treatment outcome of behavioral and nondirective parent training and to examine the usefulness of the PAI^8^ in deriving individualized treatment recommendations. | Multimodal profile | RCT | Y | N = 110 (youth with ADHD or ODD)  20.0% female  4-11 years | Families randomized to their PAI-predicted optimal intervention showed a treatment advantage of d = 0.54, 95% CI [0.17, 0.97]; for ADHD, the advantage was negligible at d = 0.35, 95% CI [–0.01, 0.78]. |
| Huang (2021) | USA | Demonstrate the clinical feasibility and technical implementation of an evidence-based, fully transparent bioinformatics pipeline for whole genome sequencing in youths with ASD. | Omics | Case series | N | N = 6 (youth with ASD)  33.3% female  3-19 years | Confirmed a portion of the key variants with Sanger sequencing and provided interpretation with consideration of patients’ clinical symptoms and detailed literature review. |
| Kuehn (2022) | USA | Illustrate one application of the idiographic approach in the context of STB^9^ research focusing on person-specific variability in associations between STBs, coping strategies, and ability to refrain from suicidal action. | Multimodal profile | Case series | Y | N = 3 (youth with failed suicide attempt and psychiatric hospital discharge)  Sex not stated  13-17 years | Individuals who report similar suicidal risk levels likely respond in individualized ways to suicidal urges necessitating personalized assessment and treatment. |
| Kusuma (2024) | Australia | Develop separate models to predict suicide attempts within a cohort at middle and late adolescence. | Big data; ML^10^ | Cohort | Y | N = 2266 (94 attempted suicide; 2172 TD^11^)  49.5% female  14-17 years | The late adolescence models performed better than the mid-adolescence models. |
| McKay (2020) | UK | Explore the thoughts of parents of children with behavioral and conduct problems regarding parenting programs and how they could be personalized. | None | Qualitative | Y | N = 42 (parent-youth dyads)  35.7% female  4-10 years | Findings point to the potential of personalized approaches to extend the reach of parenting programs to parents and children who do not currently benefit from such programs. |
| Peris (2013) | USA | Examine the feasibility and acceptability of a personalized intervention for pediatric OCD^12^ characterized by certain family profiles. | None | RCT | N | N = 20 (10 youth with OCD with standard treatment; 10 youth with OCD with positive family interactive therapy)  45.0% female  8-17 years | Families receiving standard treatment demonstrated a 40% response rate, but families receiving positive family interactive therapy demonstrated a 70% response rate. |
| Peris (2017) | USA | Examine the efficacy of a personalized intervention module designed for cases of OCD characterized by certain family profiles. | None | RCT | N | N = 62 (30 youth with OCD with standard treatment; 32 youth with OCD with positive family interactive therapy)  43.5% female  Age range not stated | Personalized treatment demonstrated a clear advantage in terms of overall response and remission rates, and reductions in functional impairment; likewise, personalized treatment outperformed standard treatment on measures of family functioning, producing significantly better reductions in symptom accommodation and family conflict. |
| Sabatello (2021) | USA | Investigate views of teens on translating genetic-based knowledge about psychiatric risks into preventive behaviors. | None | Cross-sectional | N | N = 417 (youth)  51.1% female  14-17 years | Found high interest among the population in learning about genetic and environmental factors contributing to psychiatric disorders. |
| VandeVoort (2022) | USA | Investigate effect of psycho-PGx testing in clinical decision making in treatment of MDD. | Omics | RCT | N | N = 176 (youth with MDD)  78.1% female  13-18 years | No outcome differences between TAU and treatment including PGx^13^. |
| Voss (2019) | USA | Evaluate the efficacy of a smart device, driven by artificial intelligence, for improving social outcomes of children with ASD. | Digital health data | RCT | N | N = 71 (youth with ASD)  11.3% female  0-12 years | Children receiving the intervention showed significant improvements in socialization compared with TD controls. |
| Young (2021) | USA | Evaluate whether MDD prevention programs can be optimized by matching youths to interventions specific to their psychosocial vulnerabilities. | None | RCT | N | N = 204 (youth)  56.4% female  11-18 years | Matched adolescents showed greater decreases in depressive symptoms than mismatched adolescents. |
| 1. SCL = skin conductance level  2. CD/ODD = conduct disorder / oppositional defiant disorder  3. RCT = randomized control trial  4. TAU = treatment as usual  5. ASD = autism spectrum disorder  6. ADHD = attention deficit hyperactivity disorder  7. MDD = major depressive disorder  8. PAI = Personalized Advantage Index  9. STB = suicidal thoughts and behaviors  10. ML = machine learning  11. TD = typically developing  12. OCD = obsessive-compulsive disorder  13. PGx = pharmacogenetics | | | | | | | |
