# Supplementary material for: Scoping review of precision child and youth mental health research: dwelling in possibility
Source: Front Psychiatry. 2026 Feb 9;16:1691548. doi: 10.3389/fpsyt.2025.1691548 (PMC12926772; doi:10.3389/fpsyt.2025.1691548)
Supplement: Supplementary file 7 [file Table7.docx]

**Supplementary Table 7. Details of predictive algorithm studies by first author’s last name (N=6)**

| **First Author (Publication Year)** | **Country** | **Aim** | **PCYMH Tools** | **Design** | **Secondary Analysis** | **Sample** | **Key Findings** |
| --- | --- | --- | --- | --- | --- | --- | --- |
| Eni (2020) | Israel | Compare the effectiveness of different ML^1^ algorithms for predicting the diagnosis of ASD^2^. | ML | Case-control | N | N = 72 (youth with ASD)  12.5% female  Age range not stated | A convolutional neural network yielded the best results. |
| Kusuma (2024) | Australia | Use supervised ML to prospectively predict suicide attempts in a nationally representative cohort of Australians at two developmental stages: middle (age 14-15) and late (age 18-19) adolescence. A second aim was to compare the models' features and predictive performance as this cohort ages. | Big data; ML | Cohort | Y | N = 2266 (94 youth attempted suicide; 2172 controls)  49.5% female  14-18 years | The overall best-performing model used random forests in late adolescence, with the late adolescence models generally performing better than the mid-adolescence models. |
| Lamb (2024) | USA | Examine the utility of using neurocognitive data in combination with an ML algorithm to predict client selection of strategies in a virtual environment. | None | Cohort | N | N = 50 (youth engaged in DBT^3^ skill development)  52.0% female  9-11 years | Neurocognitive data in may be used to successfully predict client outcome and increase the quality and reliability of artificially intelligent counselors and improve counselors use of client-based analytics in face-to-face and digital counseling environments. |
| Liu (2016) | China | Examine whether face scanning patterns could be used to identify children with ASD using ML. | ML | Case-control | N | N = 87 (29 youth with ASD; 58 controls)  13.8% female  4-11 years | Manifest the effectiveness and feasibility of applying the ML algorithm based on the face scanning patterns in classifying and predicting ASD. |
| Saggu (2024) | Canada | Examine the accuracy and performance of GNN^4^ ML models compared to RNN^5^, baseline conventional ML, and regression models for predicting emergency department revisits. | Big data; ML | Cohort | N | N = 4,473 (youth)  Sex not stated  Age range not stated | The GNN model outperformed both the RNN model and the best performing conventional ML model. |
| Walsh (2018) | USA | Examine whether an ML approach could produce accurate prediction of adolescent suicide attempts. | ML | Cohort | N | N = 26,055 (974 youth with failed suicide attempt; 496 youth with other self-injury; 7,059 youth with MDD^6^; 25,081 controls)  49.0% female  0-18 years | Computational models outperformed standard logistic regression in prediction of suicide risk. |
| 1. ML = machine learning  2. ASD = autism spectrum disorder  3. DBT = dialectical behavioral therapy  4. GNN = graph neural network  5. RNN = recurrent neural network  6. MDD = major depressive disorder | | | | | | | |
